# Using epidemiological evidence to forecast population need for early treatment programmes in mental health: a generalisable Bayesian prediction methodology applied to and validated for first-episode psychosis in England

**DOI:** 10.1192/bjp.2021.18

**Published:** 2021-07

**Authors:** Keltie McDonald, Tao Ding, Hannah Ker, Thandiwe Rebecca Dliwayo, David P.J. Osborn, Pia Wohland, Jeremy W. Coid, Paul French, Peter B. Jones, Gianluca Baio, James B. Kirkbride

**Affiliations:** 1 Division of Psychiatry, University College London, UK; 2 Department of Statistical Sciences, University College London, UK; 3 School of Earth and Environmental Sciences, University of Queensland, Australia; Hull-York Medical School, University of Hull, UK; 4 Mental Health Center and Psychiatric Laboratory, The State Key Laboratory of Biotherapy, West China Hospital of Sichuan University, China; 5 Faculty of Health, Psychology and Social Care, Manchester Metropolitan University, UK; 6 Department of Psychiatry, University of Cambridge, UK

**Keywords:** Psychotic disorders, epidemiology, Bayesian statistics, prediction, health services research

## Abstract

**Background:**

Mental health policy makers require evidence-based information to optimise effective care provision based on local need, but tools are unavailable.

**Aims:**

To develop and validate a population-level prediction model for need for early intervention in psychosis (EIP) care for first-episode psychosis (FEP) in England up to 2025, based on epidemiological evidence and demographic projections.

**Method:**

We used Bayesian Poisson regression to model small-area-level variation in FEP incidence for people aged 16–64 years. We compared six candidate models, validated against observed National Health Service FEP data in 2017. Our best-fitting model predicted annual incidence case-loads for EIP services in England up to 2025, for probable FEP, treatment in EIP services, initial assessment by EIP services and referral to EIP services for ‘suspected psychosis’. Forecasts were stratified by gender, age and ethnicity, at national and Clinical Commissioning Group levels.

**Results:**

A model with age, gender, ethnicity, small-area-level deprivation, social fragmentation and regional cannabis use provided best fit to observed new FEP cases at national and Clinical Commissioning Group levels in 2017 (predicted 8112, 95% CI 7623–8597; observed 8038, difference of 74 [0.92%]). By 2025, the model forecasted 11 067 new treated cases per annum (95% CI 10 383–11 740). For every 10 new treated cases, 21 and 23 people would be assessed by and referred to EIP services for suspected psychosis, respectively.

**Conclusions:**

Our evidence-based methodology provides an accurate, validated tool to inform clinical provision of EIP services about future population need for care, based on local variation of major social determinants of psychosis.

## Background

The past decade has witnessed an unprecedented transformation in public perception of mental health,^1^ with political commitment^2,3^ to address substantial disparities between physical and mental healthcare.^4^ This has begun to have an impact on mental healthcare provision, which is undergoing substantial reconfiguration in many regions, including the USA,^5^ Canada,^6^ Australia^7^ and northern Europe.^2,8,9^ One such example is early intervention in psychosis (EIP) services, which seek to provide multidisciplinary care for people with an emerging psychosis, informed by an evidence base that suggests that a longer duration of untreated psychosis is associated with less favourable health and social outcomes.^10^ These services have rapidly gained traction in several countries, including England, based on clinical evidence for their efficacy,^11^ effectiveness^11,12^ and potential cost-effectiveness^13^ over the short to medium term. Effects on longer-term outcomes, particularly in the absence of sustained intervention, are less apparent,^14^ and are a challenge to providing sustainable care models for people experiencing psychosis.

## Evidence-based public mental health

Optimising this care requires a broader evidence-based, pragmatic approach,^15^ incorporating epidemiological knowledge to aid decision-making, to effectively allocate resources to EIP services based on anticipated local need in different populations. For example, age, gender and urbanicity have been consistently associated with the incidence of psychotic disorders in the UK, Europe and potentially beyond,^16^ and their local distributions could therefore inform population-level need for treatment each year. Such population-based approaches are increasingly recognised as vital to effective public mental health, but few examples are available to inform local and national mental health policy.^15,17^

One example previously applied to population-level need for EIP care in England is known as the PsyMaptic model (Psychiatric Mapping Translated into Innovations for Care).^18,19^ Based on empirical epidemiological estimates of the incidence of first-episode psychosis (FEP) by age, gender, ethnicity and selected area-level characteristics such as deprivation, this model predicted the incidence (number and rate of new cases) in different population groups and regions based on the population structure of the 2011 Census of Great Britain. This model has subsequently informed national policy-making in the UK,^20,21^ including implementation guidelines for the landmark ‘Access and Waiting Time’ standard for EIP care in 2016,^20^ as part of the National Health Service and Department of Health's ‘Five-Year Forward View for Mental Health’,^2^ which legislated commitment to parity of esteem between physical and mental health.^22^ This standard mandated that at least 60% of all people aged 14–65 years who are referred to EIP services for ‘suspected psychosis’ must have been assessed, and where suitable, commenced a National Institute of Health and Care Excellence (NICE)-concordant,^22,23^ evidence-based^24,25^ package of EIP care within 14 days. However, although there is emerging evidence that most EIP services in England are already exceeding the waiting time target,^26^ achieving fidelity to other targets in the standard, including offering cognitive–behavioural therapy for psychosis, family interventions and supported employment and education programmes, has proved challenging,^26^ and may erode potential beneficial outcomes expected for patients available when full-fidelity EIP care is offered.^17^ A prerequisite to achieving such fidelity is that mental health service policy makers, commissioners and providers have access to timely and accurate information about future local need for EIP care, so that services can be appropriately resourced to deliver effective, optimised evidence-based care.

Nonetheless, the previously published PsyMaptic tool^18^ had limited generalisability to forecast future need for EIP care. For example, it was restricted to (a) a static denominator population, using the 2011 Census; (b) incidence data ascertained before the national introduction of EIP services in 2002; (c) social determinants of health data at a local authority level (serving large, heterogenous areas; median population in 2018: 126 678 people^27^) and (d) ICD-10 diagnostic criteria for psychotic disorders, and not the broader need for assessment and treatment generated by referrals to EIP services for suspected psychosis.^20^

## Aim

To overcome these limitations and facilitate generalisability of our methodology to other settings, we developed a new, population-based prediction model applied to psychosis care in England, validated against observed national routine data. Our methodology forecasted small-area need for EIP provision up to 2025 to inform the ‘NHS Long-Term Plan’^28^ and ‘Mental Health Implementation Plan in England’,^29^ which have recently reaffirmed and strengthened their commitment to EIP care, taking into account projected changes in the population at risk in England over the next 5 years.

## Method

We followed the transparent reporting of multivariable prediction modell for individual prognosis or diagnosis reporting guidelines for prediction modelling (Supplementary Box 1 available at https://doi.org/10.1192/bjp.2021.18).

### Model development

#### Seed data

Empirical incidence data were pooled from the three largest epidemiological catchment area studies of psychotic disorders in England in the past 25 years, which used similar methodologies, as described in detail elsewhere^30–33^ (Supplementary Section 1): the Aetiology and Ethnicity in Schizophrenia and Other Psychoses (ÆSOP),^30^ the East London First Episode Psychoses (ELFEP)^32,33^ and Social Epidemiology of Psychoses in East Anglia (SEPEA)^31^ studies.

#### Outcome

These studies included incidence data on all people aged between 16–64 years who presented to services (including EIP^31^) in each catchment area for suspected psychosis, and who later met research-based criteria for a diagnosis of for non-affective psychotic disorder (ICD-10 codes F20–29), affective psychotic disorder (ICD-10 codes F30–33) or substance-induced psychotic disorder (ICD-10 code F1X.5). Individuals with an organic basis to their disorder or profound intellectual disability were excluded.

#### Predictors

Participants were linked to their small-area neighbourhood of residence at first presentation (ÆSOP/ELFEP: 2001 Census Area Statistics wards; SEPEA: 2011 Census merged wards). For each ward, we obtained measures of relevant socioenvironmental data, guided by previous models and the wider literature,^18,34,35^ including population density; social fragmentation; own-group ethnic density; multiple deprivation; inequality and the gender-specific, age-standardised prevalence of self-reported past-year cannabis use in the population (Supplementary Section 2). We stratified our seed data-set by age group (16–17, 18–19, 20–24, 25–29, 30–34, 35–39, 40–44, 45–49, 50–54, 55–59 and 60–64 years), gender and self-assigned ethnicity (based on *a priori* evidence about risk:^34,36^ White British, Irish, Gypsy and Traveller; White other; Black Caribbean and Black other; Black African; Indian; Pakistani; Bangladeshi; mixed; and all other ethnicities) at ward level. We recorded the count of FEP cases and person-years at risk in each strata, estimated from the nearest decennial census to the case ascertainment period (Supplementary Section 3). Ward- and regional-level covariates were appended to this data-set.

#### Model building

**Fig. 1:**
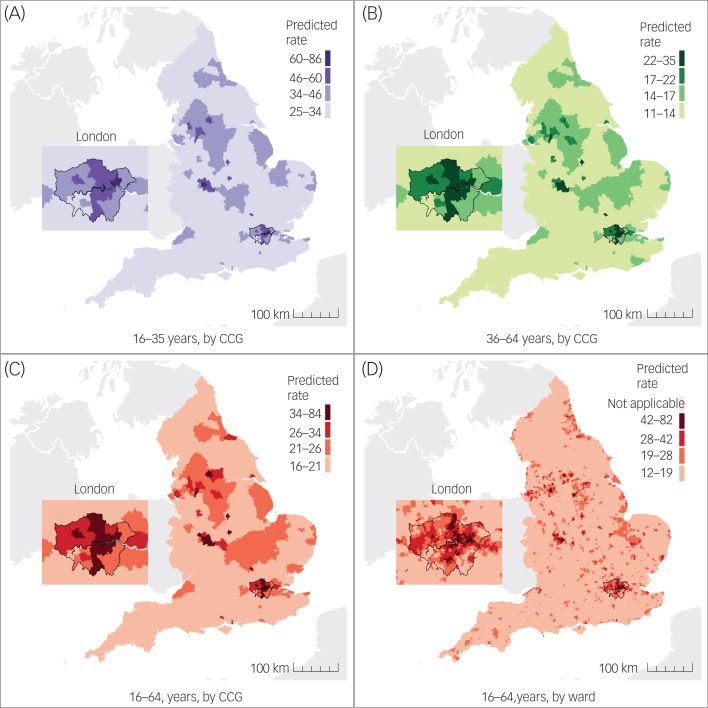
Visualisation of predicted incidence rates of FEP per 100 000 person-years by age and geographical level in England, 2020. Predicted incidence rate per 100 000 person-years at the CCG level for people aged (A) 16–35 years, (B) 36–64 years, (C) 16–64 years and (D) at ward level for people aged 16–64 years. Predictions were not produced for five Census merged wards (Isles of Scilly [one ward] and all four wards within City of London) because of inaccurate population estimates, and values are not shown in (D). CCC, Clinical Commissioning Group; FEP, first-episode psychosis.

We modelled the count of new (incidence) cases of psychotic disorders by using Poisson regression in a Bayesian framework. A Poisson distribution was preferred to a negative binomial distribution as there was no evidence of overdispersion within the seed data-set (Supplementary Table 1). The Bayesian approach allowed us to combine this data-set with *a priori* knowledge about the epidemiology of psychotic disorders, specified as prior probability distributions on the covariates to be estimated, informed by published evidence^34–37^ (Supplementary Section 4, Figs 1–3 and Tables 2–5). We fitted our models by using integrated nested Laplace approximation^38^ to estimate the posterior distribution for the relevant parameters. Then, to fully characterise the uncertainty in the estimates and the predictions, we used Monte Carlo simulation to randomly sample 1000 values from the joint posterior distributions of each model parameter, simultaneously accounting for any correlation present between parameters in the model. This allowed us to capture the full uncertainty around the effect of each potential covariate on FEP incidence rates, which we expressed as posterior relative risks with their 95% credible interval estimates.

Initial modelling suggested that ethnic density (relative risk 1.001, 95% CI 0.998–1.005) and inequality (relative risk 0.90, 95% CI 0.50–1.62) were not associated with FEP incidence in our seed data-set, and were removed from further consideration. From the remaining covariates, we considered six *a priori* candidate models. We included age group, gender, their interaction and ethnic group in all models, with different permutations of deprivation, population density, social fragmentation and cannabis use fitted in each model. Person-years at risk were treated as an offset in the models.

### Out-of-sample prediction

#### Denominator and socioenvironmental data

We estimated the projected population at risk, aged 16–64 years, for all Census merged wards in England (*N* = 7678) between 2017–2025, stratified by age group, gender and ethnicity (as before). Projected population estimates up to 2025 were obtained from demographic forecasts with an established methodology,^39,40^ using 2011 Census data as a baseline, accounting for year-on-year changes in birth and death rates, immigration and emigration flows, and including assumptions about the impact of Brexit beyond 2020^40^ (Supplementary Section 5). Population projections for the City of London (four wards) and Isles of Scilly (one ward) were excluded because of substantial overestimation of the ward population, when compared with Office for National Statistics 2017 mid-year population estimates.^41^ For socioenvironmental covariates, we used the latest available routine data at the time of model development for deprivation (2015),^42^ social fragmentation (2011), population density (re-estimated for each year between 2017–2025, based on ward-level projected populations) and regional cannabis prevalence (2014)^43^ (Supplementary Section 2).

#### FEP prediction

Posterior relative risk coefficients from each model were applied to the projected denominator data in a given year (2017–2025) at ward level, to estimate the predicted number of incident FEP cases in each age-gender-ethnic stratum in each ward. Within our Bayesian framework, we performed 1000 model simulations, sampling from the posterior distribution to yield the mean predicted FEP cases per stratum, and corresponding 95% confidence interval, derived from the 2.5% and 97.5% quantiles. This allowed us to aggregate prediction data to Clinical Commissioning Group (CCG) level and the national level by gender, broad age group (16–64, 16–35 and 36–64 years; consistent with those most relevant to EIP care in England) and ethnicity for any given year (2017–2025) (Supplementary Section 6). CCGs are autonomous, clinically led, statutory National Health Services (NHS) bodies responsible for the planning and commissioning of health services in local areas.^44^

### Model validation

#### Apparent validation

Apparent validity of the six candidate models was evaluated with the deviance information criterion (DIC), with a smaller value indicating better fit.^34,45^

#### External validation

To externally validate our models, we compared predicted FEP data based on the 2017 denominator population, with observed national data available from NHS Digital's Mental Health Services Dataset (MHSDS) in the closest (financial) year available (April 2017 to March 2018; henceforth ‘2017’). Comparisons were matched for the EIP age range served by each CCG in 2017 (Supplementary Section 7).^46^ The MHSDS records data from all health records for people in contact with mental health services in England. From this data-set, we extracted data for each CCG in England (*N* = 207) on the total number of new people (a) referred to mental health services for suspected psychosis; (b) accepted for assessment by EIP services; (c) who commenced EIP treatment for FEP and (d) who were probable FEP cases, based on empirical evidence that approximately 14% of those who commenced treatment for FEP in EIP services do not go onto to fulfil operationalised criteria for ICD-10 psychotic disorder.^31^ Those not meeting criteria for probable FEP in categories a–c implicitly included people meeting the clinical high risk for psychosis (CHR-P) criteria, who are not currently separately disaggregated within the MHSDS data-set. Formal definitions of each of these nested levels, henceforth referred to as referred/assessed/treated/probable FEP case-loads, are given in Supplementary Section 7.

We inspected external validity at the CCG level by estimating correlation coefficients (*r*) between observed and predicted probable FEP counts, the *r*^2^ statistic and root-mean-square error (RMSE), with higher *r*^2^ and lower RMSE indicating better model fit. We inspected calibration plots of predicted versus observed FEP counts and compared the difference in predicted and observed FEP counts, expressed as a rate per 100 000 person-years at the CCG level. At the national level, we compared the difference between predicted and observed case-loads, expressed as a percentage, for the total count and by broad age group, gender and ethnic group. Choice of our best-fitting model was determined by consensus agreement between our authorship group, after evaluation of all apparent and external validation metrics.

#### Missing data

We excluded two CCGs from external validation (NHS Birmingham & CrossCity CCG and NHS Sandwell and West Birmingham CCG) because of problems with data validity in the MHSDS during this reporting period (C. Money, personal communication, 2019). These CCGs were reinstated for prediction forecasting (see below) between 2019 and 2025. MHSDS data were complete with respect to national- and CCG-level totals and by broad age group (16–35 and 36–64 years). Ten (0.1%) observed probable FEP cases were missing information on gender, and were excluded from gender-specific validation. Ethnicity data were missing for 820 (10.2%) observed probable FEP cases, which would have affected model validity by ethnicity without imputation, which we handled via imputation in sensitivity analyses (Supplementary Section 8).

### Forecasting

Our best-fitting model was used to forecast predicted probable FEP case-loads in England between 2019–2025, using the relevant denominator projections for each year. Predictions and their 95% confidence intervals, stratified by broad age group, gender and ethnicity, were aggregated to 2019 CCG and national levels for dissemination. We also forecasted new referred, assessed and treated case-loads (implicitly including CHR-P individuals) per annum by multiplying predicted probable FEP case-loads by the ratios derived from the observed data in the 2017 MHSDS (Supplementary Section 9). All prediction forecasts were expressed as incidence counts, and rates per 100 000 persons-years.

### Software

Data-set generation and validation were performed in Stata version 15 for Windows,^47^ prediction modelling was conducted with the integrated nested Laplace approximation package in R version 3.5 for Windows (R Foundation, Austria; see https://www.r-project.org/),^38^ and out-of-sample prediction using matrix multiplication was performed in R.^48^

### Ethics

The authors assert that all procedures contributing to this work comply with the ethical standards of the relevant national and institutional committees on human experimentation, and with the Helsinki Declaration of 1975, as revised in 2008. All procedures involving human patients were approved by NHS Health Research Authority (approval reference 19/HRA/0145). Consent was not required for this study, which was based on secondary use of aggregated, anonymised data from the ÆSOP, ELFEP and SEPEA studies and similarly aggregated NHS MHSDS data.

## Results

### Seed data sample characteristics

From our seed data-set, we included 1638 cases of FEP from 4 515 379 person-years at risk (Table 1). As typical of FEP incidence samples,^34^ most cases were male (62%), younger than 30 years old at first contact (ÆSOP 54%, ELFEP 57%) and with an overrepresentation of participants from Black and minority ethnic backgrounds relative to the local at-risk population (varying from 24% in SEPEA^49^ to 77% in ELFEP^33^).

**Table 1:** Summary of FEP data from the ÆSOP, ELFEP and SEPEA studies

	Cases, *n* (%)				
	ÆSOP^a^	ELFEP^a^	SEPEA^a^	Total cases	Total person-years
Person-years at risk	1 653 020	840 587	2 021 772	–	4 515 379
Total	551 (33.6)	428 (26.1)	659 (40.2)	1638 (100.0)	–
Age group, years					
16–17	22 (4.0)	–	77 (11.7)	99 (6.0)	226 029 (5.0)
18–19	51 (9.3)	37 (8.6)	110 (16.7)	198 (12.1)	303 055 (6.7)
20–24	119 (21.6)	91 (21.3)	232 (35.2)	442 (27)	851 113 (18.8)
25–29	107 (19.4)	116 (27.1)	142 (21.6)	365 (22.3)	890 523 (19.7)
30–34	90 (16.3)	63 (14.7)	86 (13.1)	239 (14.6)	860 693 (19.1)
35–39	60 (10.9)	43 (10.1)	12 (1.8)	115 (7.0)	418 069 (9.3)
40–44	37 (6.7)	28 (6.5)	–	65 (4.0)	257 386 (5.7)
45–49	21 (3.8)	23 (5.4)	–	44 (2.7)	208 330 (4.6)
50–54	18 (3.3)	11 (2.6)	–	29 (1.8)	198 414 (4.4)
55–59	14 (2.5)	10 (2.3)	–	24 (1.5)	157 696 (3.5)
60–64	12 (2.2)	6 (1.4)	–	18 (1.1)	144 071 (3.2)
Gender					
Female	231 (41.9)	169 (39.5)	222 (33.7)	622 (38.0)	2 240 659 (49.6)
Male	320 (58.1)	259 (60.5)	437 (66.3)	1016 (62.0)	2 274 720 (50.4)
Ethnicity					
White British, Irish and Traveller	262 (47.6)	98 (22.9)	498 (75.6)	858 (52.4)	3 288 403 (72.8)
White other	32 (5.8)	56 (13.1)	60 (9.1)	148 (9.0)	354 296 (7.8)
Black Caribbean	126 (22.9)	70 (16.4)	10 (1.5)	206 (12.6)	168 884 (3.7)
Black African	69 (12.5)	67 (15.7)	22 (3.3)	158 (9.7)	172 731 (3.8)
Indian	10 (1.8)	25 (5.8)	2 (0.3)	37 (2.3)	108 359 (2.4)
Pakistani	8 (1.5)	16 (3.7)	17 (2.6)	41 (2.5)	69 373 (1.5)
Bangladeshi	1 (0.2)	64 (15.0)	6 (0.9)	71 (4.3)	114 754 (2.5)
Mixed	21 (3.8)	18 (4.2)	18 (2.7)	57 (3.5)	97 289 (2.2)
Other	22 (4.0)	14 (3.3)	26 (4.0)	62 (3.8)	141 290 (3.1)

FEP, first-episode psychosis; ÆSOP, Aetiology and Ethnicity in Schizophrenia and Other Psychoses study; ELFEP, East London First Episode Psychoses study; SEPEA, Social Epidemiology of Psychoses in East Anglia study.

a. Study age ranges: ÆSOP, 16–64 years; ELFEP, 18–64 years; SEPEA, 16–35 years.

### Apparent validation

Apparent validation of our models suggested that inclusion of area-level predictors (Table 2, models 2–6, DIC 11 531.88–11 539.75) improved model fit over a model fitted solely with individual-level covariates (model 1: DIC 11 614.04); DIC values were equivocal for models 2 and 4–6 (Table 2). Full model parameter specifications (Supplementary Table 6) showed elevated posterior relative risk for young men and several ethnic minority groups, particularly people of Black Caribbean (relative risk_model 4_ 4.80, 95% CI 4.08–5.63) and Black African (relative risk_model 4_ 3.33, 95% CI 2.78–3.97) ethnicity, consistent with the available literature.^34^ Population density was weakly associated with FEP incidence (relative risk_model 5_ 1.06, 95% CI 0.99–1.12). Deprivation showed a non-linear relationship with FEP incidence, with exponentially higher rates at increasingly levels of deprivation (Supplementary Fig. 3). In contrast to expectations, a 1-s.d. increase in social fragmentation was associated with lower incidence (relative risk_model 4_ 0.92, 95% CI 0.87–0.97). As expected, and driven by prior information,^35^ lifetime self-reported cannabis use was strongly associated with psychosis incidence (relative risk_model 4_ 1.41, 95% CI 1.16–1.72).

**Table 2:** Apparent and external validity of six candidate Bayesian Poisson regression models

	Model 1	Model 2	Model 3	Model 4	Model 5	Model 6
Covariates	Age, gender, age×gender, ethnicity	Model 1 plus deprivation, social fragmentation	Model 1 plus deprivation, population density	Model 2 plus cannabis use	Model 4 plus population density	Model 2 plus population density
Apparent validity						
Model DIC	11 614.04	11 533.86	11 539.75	11 533.72	11 531.93	11 531.88
External validity						
National level						
Observed cases,^a^ *n*	8038	8038	8038	8038	8038	8038
Predicted cases, *n* (95% CI)^b^	8984 (8497 to 9460)	8137 (7642 to 8637)	8362 (7907 to 8851)	8112 (7623 to 8597)	8187 (7722 to 8707)	8205 (7716 to 8763)
Difference, *n* (%)	946 (11.77)	99 (1.24)	324 (4.04)	74 (0.92)	149 (1.85)	167 (2.08)
CCG level (*n* *=* 205)^c^						
RMSE	23.56	20.95	21.55	20.94	21.29	21.32
*R*^2^	0.49	0.54	0.54	0.54	0.53	0.53
Correlation coefficient, *R*	0.70	0.74	0.74	0.74	0.73	0.73
Calibration slope, *β* (s.e.)	0.66 (0.05)	0.64 (0.04)	0.69 (0.04)	0.64 (0.04)	0.64 (0.04)	0.64 (0.04)
Calibration intercept	18.08	14.90	14.21	14.84	15.00	15.03
Rate difference per 100 000 person-years between predicted and observed, *n* (%)^d^						
<−20	13 (6.34)	15 (7.32)	14 (6.83)	15 (7.32)	15 (7.32)	15 (7.32)
−20 to −11	24 (11.71)	21 (10.24)	22 (10.73)	22 (10.73)	23 (11.22)	23 (11.22)
−10 to 10	109 (53.17)	123 (60.00)	122 (59.51)	123 (60.00)	121 (59.02)	121 (59.02)
11 to 20	35 (17.07)	35 (17.07)	35 (17.07)	34 (16.59)	35 (17.07)	35 (17.07)
>20	24 (11.71)	11 (5.37)	12 (5.85)	11 (5.37)	11 (5.37)	11 (5.37)

DIC, deviance information criterion; RMSE, root-mean-square error; MHSDS, Mental Health Services Dataset; CCG, Clinical Commissioning Group.

a. Observed data from NHS Digital MHSDS for the financial year April 2016 – March 2017.

b. Predicted data were age-matched to observed MHSDS data, according to the corresponding age range served by each CCG as reported in the Early Intervention in Psychosis Network national audit for the same period (see Supplementary Section 7 for further details). Not all CCGs served the entire population aged 16–64 years, so the figures in this table should not be interpreted as total predicted cases in England for a given stratum. For total predicted case-load sizes in England, for 2019–2025, see Supplementary Table 10.

c. Out of 207 CCGs, 205 were included in validation statistics; two CCGs were excluded because of reliability concerns over the observed MHSDS data.

d. Number and percentage of CCGs where difference between the observed and predicted incidence rate (per 100 000 person-years) fell within the category range shown.

### External validation

#### National level

In 2017, 22 803 new referrals for suspected psychosis were recorded in the MHSDS across the 205 CCG included for validation; 89.9% (*n* = 20 492) were subsequently accepted for EIP assessment, 35.2% (*n* = 9346) commenced EIP treatment and 35.2% (*n* = 8038; 86% of the treated case-load) met our definition of probable FEP. For the same period, model 4 (individual covariates, deprivation, social fragmentation and cannabis prevalence) predicted 8112 FEP cases per annum (95% CI 7623–8597), giving the lowest error of any model at the national level (*n* = +74, +0.92%), compared with the observed probable FEP case-load (Table 2).

All models performed similarly against national observed probable FEP case-loads stratified by major age group, gender and ethnic group (Supplementary Tables 7–9). Model 4 (Table 3), for example, showed good accuracy by gender (Table 3) and ethnicity after imputation for missing observed data (Supplementary Section 8 and Table 9). Nonetheless, all models tended to underpredict FEP case-loads aged 16–35 years (e.g. model 4: *n* = −541, −9.2%), and, correspondingly, overpredicted case-loads aged 36–64 years (e.g. model 4: *n* = +615, +29.0%) compared with observed probable FEP case-loads in the MHSDS data-set.

**Table 3:** Predicted counts and incidence rates of new FEP (model 4) and corresponding observed data in the MHSDS in 2017^a^

		Observed probable FEP (MHSDS)			Predicted FEP (PsyMaptic-A model 4)			Difference (predicted minus observed)	
	2017 Population at risk^b^	Cases		Rate	Cases		Rate (95% CI)		
		*n*	%		*n* (95% CI)	%		*n*	%
Total, age-matched	32 125 420	8038	100.00	25.02	8112 (7623–8597)	100.00	25.25 (23.73–26.76)	74	0.92
Age, years									
16–35	14 333 850	5919	73.64	41.29	5378 (5078–5695)	66.30	37.52 (35.42–39.73)	−541	−9.15
36–64	17 791 569	2119	26.36	11.91	2734 (2424–3053)	33.70	15.37 (13.63–17.16)	615	29.02
Gender									
Female	16 046 762	3240	40.31	20.19	3178 (2899–3467)	39.18	19.8 (18.06–21.61)	−62	−1.92
Male	16 078 658	4790	59.59	29.79	4934 (4597–5284)	60.82	30.69 (28.59–32.86)	144	3.00
Unknown^c^		10	0.12						
Ethnicity									
White British, White Irish and Traveller	23 997 617	4684	58.27	19.52	4570 (4235–4907)	56.33	19.04 (17.65–20.45)	−114	−2.44
White other	2 474 534	531	6.61	21.46	767 (652–897)	9.45	30.99 (26.36–36.26)	236	44.43
Black Caribbean	538 118	370	4.60	68.76	538 (471–619)	6.63	99.95 (87.53–115.11)	168	45.36
Black African	787 915	323	4.02	40.99	593 (496–688)	7.31	75.22 (62.93–87.31)	270	83.49
Indian	1 060 364	144	1.79	13.58	281 (202–386)	3.47	26.54 (19.09–36.37)	137	95.43
Pakistani	705 815	237	2.95	33.58	370 (272–505)	4.56	52.39 (38.52–71.49)	133	56.03
Bangladeshi	278 900	109	1.36	39.08	125 (96–156)	1.54	44.77 (34.52–55.82)	16	14.56
Mixed	728 404	249	3.10	34.18	489 (368–615)	6.03	73.33 (56.43–94.26)	240	96.36
Other	1 553 752	570	7.09	36.69	380 (295–476)	4.68	27.38 (21.57–34.91)	−190	−33.42
Unknown^c^		820	10.20					−820	−10.20

Rate shows the incidence rate per 100 000 person-years. FEP, first-episode psychosis; MHSD, Mental Health Services Dataset; CCG, Clinical Commissioning Group.

a. Predicted data were age-matched to observed MHSDS data, according to the corresponding age range served by each CCG as reported in the Early Intervention in Psychosis Network national audit for the same period (see Supplementary Section 7 for further details). Not all CCGs served the entire population aged 16–64 years, so the figures in this table should not be interpreted as total predicted cases in England for a given stratum. For total predicted case-load sizes in England, for 2019–2025, see Supplementary Table 10.

b. Reflects the total population according to age ranges accepted by individual CCGs.

c. Includes unknown, indeterminate and invalid values.

#### CCG level

At the CCG level, fit indices (Table 2) suggested that models 2–6 (with area-level covariates) provided a better fit to the data than model 1 (individual-level covariates only). Differences in fit indices between models for models 2–6 were small, although model 4 was associated with lowest RMSE and joint-highest *r^2^* correlation coefficients (with models 2 and 3). For all models, calibration slopes were positive (e.g. model 4: *β* = 0.64; s.e. 0.04; Table 2**,** Supplementary Fig. 4), indicating good agreement between observed and predicted probable FEP case-loads. For model 4, 60% (123 of 205) of predicted probable FEP case-loads at the CCG level were within ±10 cases per 100 000 person-years of the observed case-loads, with 87% (179 of 205) within ±20 cases per 100 000 person-years (Table 2).

#### Candidate model selection

Models 2–6 generally yielded similar apparent and external validation indices, but we interpreted model 4 as performing most consistently across all parameters (Tables 2 and 3). Visual inspection of maps of observed and predicted FEP case-loads for CCGs in 2017 indicated good apparent validity (Supplementary Fig. 5), although some spatial variation in differences remained evident, particularly in South England (Supplementary Fig. 6c).

### Forecasting

Using model 4, we forecasted referred, assessed, treated and probable FEP case-loads in England between 2019 and 2025, based on population projections for each year (Supplementary Fig. 7 and Table 10). For 2020, our model predicted a new referred case-load of 24 499 (95% CI 22 930–25 991), an assessed case-load of 22 033 (95% CI 20 622–23 375), a treated case-load of 10 516 (95% CI 9843–11 157) and a probable FEP case-load of 9066 (95% CI 8485–9618) aged 16–64 years (predicted probable FEP incidence rate of 25.2 cases per 100 000 person-years, 95% CI 23.6–26.8). Between 2019 and 2025, predicted treated case-loads increased by 6.2%, on average, but we could not exclude the possibility of no change or a small decline in case-loads over time (95% CI −3.2% to 15.6%; Supplementary Table 10).

Forecasted FEP incidence varied geographically (for example, in 2020, see Fig. 1), with higher rates in CCGs serving most major conurbations, including Greater London, Greater Manchester, Bradford and Birmingham and the Midlands (Supplementary Table 1^1^). We also predicted that some CCGs with relatively low predicted incidence rates would have large predicted counts of cases (i.e. new case-load sizes), given the large at-risk populations and geographical areas they served (Supplementary Table 1^2^). Complete forecast prediction data at CCG and national levels for 2019–2025 is available at www.psymaptic.org via an open-access web visualisation platform and downloadable data-sets.

## Discussion

### Principal findings

We developed and validated a population-level prediction model to forecast need for mental healthcare, here applied to referral to, assessment by and treatment for suspected psychosis in EIP services in England, using robust epidemiological data and small-area population projections. Our best-fitting model yielded acceptable validity against observed routine data on probable FEP at the CCG level in England for 2017, from which we extrapolated predictions up to 2025 based on small-area population forecasts. Our model also forecasted additional levels of need generated by referred, assessed and treated case-loads to provide a comprehensive, evidence-based tool for EIP planning, consistent with the timeframe of the ‘NHS Long-Term Plan’^28^ and ‘Mental Health Implementation Plan’.^29^

### Strengths and weaknesses

Our model has a number of strengths. We synthesised robust epidemiological data on psychosis risk, prior knowledge and small-area variation in social and environmental factors, to validate an *a priori* set of candidate models against national routine data at the CCG level. Using Bayesian inference to model uncertainty around these predictions allowed us to forecast expected need for psychosis care in England up to 2025, within tolerable parameter estimates.

Prediction models are influenced by their underlying assumptions and limitations. Four issues are of particular note. First, our seed data included incident cases from approximately 10% of 2011 Census merged wards (*n* = 751 of 7678), covering 14.5% of CCGs in England. Predictions could be less accurate in areas that have very different sociodemographic or socioeconomic characteristics from those in our seed data-set regions. However, our best-fitting model displayed acceptable external validity to a national data-set at the CCG level, for total, gender- and ethnicity-specific case-loads in people aged 16–64 years. Second, our models under- and overestimated probable FEP case-loads in people aged 16–35 and 36–64 years, respectively, compared with observed data. This may have arisen from inclusion of epidemiological data in our seed data-set collected before the introduction of EIP services in England;^30,33^ there is some evidence that age at first contact with mental health services may have fallen since their introduction.^31^ Third, external validity of our models was reliant on the reliability and validity of observed FEP data from the MHSDS. Sensitivity analyses allowed us to validate our models in the presence of missing observed data on ethnicity. Uncertainty in either our predictions or the observed data could explain deviance in our calibration plots at the CCG level. Since the MHSDS only provides information on who commenced EIP treatment, and not the subset of individuals who met diagnostic criteria for FEP, we revised observed treated case-loads downward by 14% to obtain a comparable FEP sample, in line with empirical evidence.^31^ This highlights the importance of recording reliable symptomatic and likely diagnostic outcomes in routine practice. Future models should include explicit predictions of people who meet CHR-P criteria for psychosis, implicitly included in our forecasts of new referred, assessed and treated case-loads per annum, but who could not be disaggregated because there is currently no provision to identify these groups separately in the MHSDS, upon which our forecasts were based. Fourth, our models did not account for predicted need in people aged <16 years (although absolute FEP incidence is rare^50^), or homeless or prison populations.

### Implications

Our Bayesian population-level prediction model could be readily adapted and updated to forecast need beyond 2025, in other countries or for other disorders, where sufficiently reliable risk estimates and denominator data are available. Application to other countries, particularly the devolved nations in the UK, is both vital for public mental health and also offers the opportunity to establish the robustness of our models to populations with potentially different distributions of underlying risk factors for psychosis.^16^ Applied to FEP in England, our model provides healthcare commissioners with a decision-making tool to support the allocation of finite resources for EIP services, based on forecasts of need likely to arise in local populations, enhancing an earlier, more limited tool.^18^ Providing commissioners with access to evidence-based forecasts of such need should help optimise and fully implement NICE-concordant care for psychosis.^20,22^ This would provide the foundation for realising the demonstrable, evidence-based improvements in clinical and social outcomes for individuals experiencing FEP, as well as the cost-effectiveness benefits on which they were established.^24,25,51,52^ Moreover, optimal resource allocation in one mental health domain should propagate benefits to other areas of care for which mental health commissioners and planners are responsible. Our predictions also allow care providers to understand the likely sociodemographic and environmental characteristics of their case-loads, which may be useful for providing locally and regionally sensitive modes of care delivery across diverse communities.

Overall, we have developed and validated a generalisable, translational tool that used epidemiological data to forecast accurate estimates of future incidence of first-episode mental health disorders in different communities. The tool currently informs recently-updated NICE guidelines in England for EIP service provision.^53^ Our prediction methodology could be readily generalised to other settings or mental health problems underpinned by a precise, reliable epidemiology. Priorities here would include extension to predict people at clinical high risk of psychosis, ongoing recalibration in England, and extension and validation to other countries. Applied to psychosis care in England, our platform provides commissioners and providers with an open-access, evidence-based toolkit to optimise need for EIP care as and when it arises.

## Data Availability

All PsyMaptic-A prediction data are freely available at www.psymaptic.org. We will share the online platform and the results from this study with NHS England, Clinical Commissioning Groups and national, regional and local EIP service leads for patient benefit. Because of the aggregated nature of the data included in this study, it is not possible to directly contact participants from the original studies included in our seed data-set.

## Supplementary material

For supplementary material accompanying this paper visit http://dx.doi.org/10.1192/bjp.2021.18. click here to view supplementary material
