# Supplementary material for: Using epidemiological evidence to forecast population need for early treatment programmes in mental health: a generalisable Bayesian prediction methodology applied to and validated for first-episode psychosis in England
Source: Br J Psychiatry. 2021 Jul;219(1):383–91. doi: 10.1192/bjp.2021.18 (PMC7611597; doi:10.1192/bjp.2021.18)
Supplement: Supplementary file 1 [file S0007125021000180sup001.docx]

Supplemental Materials

Forecasting population need for early treatment programs in mental health using epidemiologic evidence: a generalisable Bayesian prediction methodology applied and validated for first episode psychoses in England.

[1. Seed dataset: Case ascertainment details 3](#_Toc52436687)

[2. Seed dataset: Socioenvironmental data details 3](#_Toc52436688)

[3. Seed dataset: Denominator details 3](#_Toc52436689)

[4. Prior distribution specifications 3](#_Toc52436690)

[5. Projected denominator data for England up to 2025 5](#_Toc52436691)

[6. FEP prediction: Further details 5](#_Toc52436692)

[7. Model validation: Further details of observed caseload definitions 6](#_Toc52436693)

[8. Sensitivity analyses to impute missing observed probable FEP data on ethnicity during external model validation against MHSDS data, 2017 7](#_Toc52436694)

[9. Prediction forecasting, 2019-2025: Forecasting various prediction types 8](#_Toc52436695)

[10. Supplemental References 9](#_Toc52436696)

[Supplemental Box 1. TRIPOD Checklist 10](#_Toc52436697)

[Supplemental Figure 1. Graphical representation of the prior distribution for the effect of male sex on psychosis risk in a single age group. 15](#_Toc52436698)

[Supplemental Figure 2. Graphical representation of the prior distribution included for lifetime prevalence of cannabis use on psychosis risk. 16](#_Toc52436699)

[Supplemental Figure 3. Plot showing the incidence rate per 100,000 by deprivation quantile among White British, Irish, and Traveller, females ages 16-17 years for Model 4. 17](#_Toc52436700)

[Supplemental Figure 4. Calibration plots of observed versus predicted counts of FEP cases in 205 CCGs for Models 1-6. 18](#_Toc52436701)

[Figure 5. Visualisation of (A) predicted FEP caseloads, and (B) observed probable FEP caseloads, by CCG in England, 2017 19](#_Toc52436702)

[Supplemental Figure 6. Visualisations of (A) predicted rate of FEP per 100,000, (B) observed rate of probable FEP per 100,000, and (C) differences between predicted and observed caseload as a rate per 100,000 person-years, by CCG in England, 2017. 20](#_Toc52436703)

[Supplemental Figure 7. Predicted count of annual new referred, assessed, treated and probable FEP caseloads in England between 2019-2025, by broad age group. 21](#_Toc52436704)

[Supplemental Table 1. Summary of distribution of cases across all strata of the seed data.^a^ 22](#_Toc52436705)

[Supplemental Table 2. Incidence rates of psychosis by age and sex derived from Hardoon et al. (12) used to derive prior distributions in Bayesian models. 23](#_Toc52436706)

[Supplemental Table 3. Prior distributions included in Bayesian models for each age-sex stratification, based on a normal distribution. 24](#_Toc52436707)

[Supplemental Table 4. Incidence rates of psychosis by major ethnic group derived from Bourque et al. (13) used to derive prior distributions in Bayesian models. 25](#_Toc52436708)

[Supplemental Table 5. Sex-specific prevalence of self-reported cannabis use in the adult population in each region of England, from the Adult Psychiatric Morbidity Survey 2014 (10). 26](#_Toc52436709)

[Supplemental Table 6. Posterior relative risks with corresponding 95% intervals for parameters from candidate models 1-6. 27](#_Toc52436710)

[Supplemental Table 7. Summary of predicted and observed counts of new FEP cases in 2017 for England, by age group, sex, and ethnicity (Models 1-6)^a^. 29](#_Toc52436711)

[Supplemental Table 8. Summary of differences between predicted and observed total counts of new FEP cases in England, by age, sex, and ethnicity (Models 1-6). 30](#_Toc52436712)

[Supplemental Table 9. Sensitivity analysis to evaluate predictive accuracy of Model 4 under different imputation scenarios to account for missing observed FEP data by ethnicity. 31](#_Toc52436713)

[Supplemental Table 10. Predicted referred, assessed treated and probable FEP caseloads in England, by age group between 2019-2025 32](#_Toc52436714)

[Supplemental Table 11. Twenty highest and lowest incidence rates of predicted FEP and treated caseloads in EIP services in 2020 at CCG level. 33](#_Toc52436715)

[Supplemental Table 12. Twenty highest and lowest counts of predicted FEP and treated caseloads in EIP services in 2020 at CCG level. 34](#_Toc52436716)

# Seed dataset: Case ascertainment details

ÆSOP identified new cases of FEP in persons aged 16-64 years in Bristol (1997) over 9 months, and Southeast London and Nottingham over 2 years (1997-1999). ELFEP identified new cases of FEP in persons aged 18-64 in Newham (1996-1998), and Tower Hamlets and Hackney (1998-2000) over 2 years. Both studies ascertained cases from all potential secondary mental healthcare services in the relevant catchment areas, including private care. SEPEA identified new FEP cases aged 16-35 years old referred to EIP services in East Anglia over 3.5 years (2009-2013). For further details, see Kirkbride et al. (1–3).

FEP cases were defined as people who met standardised research criteria (ÆSOP/ELFEP: Schedule for Clinical Assessments in Neuropsychiatry [SCAN](4); SEPEA: Operationalised Criteria for Psychotic Illness [OPCRIT] (5)) for a diagnosis of International Classification of Disease, tenth revision [ICD-10] non-affective psychotic disorder (F20-29), affective psychotic disorder (F30-33) or substance-induced psychotic disorder (F1X.5). Individuals with an organic basis to their disorder, or profound learning difficulty were excluded.

# Seed dataset: Socioenvironmental data details

We used the following sources to define socioenvironmental variables in the seed dataset. Census-based data were derived from the temporally closest decennial Census of Great Britain (2001 or 2011), and included the following measures estimated at “Census merged ward” level: (i) population density, as the number of people per hectare; (ii) social fragmentation, based on Congdon’s social fragmentation index (6) to produce a sum-score of four z-standardised indicators, including the proportion of single person households, unmarried persons, annual residential turnover and privately renting households, and (iii) own-group ethnic density, to estimate the proportion of a participant’s own ethnic group living in each ward. Using the temporally-closest Indices of Deprivation (ÆSOP/ELFEP: 2004 (7); SEPEA: 2015 (8)), we obtained (iv) a measure of the index of multiple deprivation [IMD] at ward level. Since IMD scores are published at “lower super output area” [LSOA] (smaller than Census merged wards) and are not directly comparable between different time points (i.e. 2004, 2015), our IMD variable was based on rank position of each LSOA by IMD in England. For a given Census merged ward, we estimated its average LSOA rank, weighted proportionally to the geographical area contribution of each LSOA to the ward. We then created a percentile distribution for all wards in England, by average IMD rank position, and extracted this for the Census merged wards in our study. The Census merged wards included in our seed data spanned the total distribution of IMD ranks in England (1-100^th^ percentile). To estimate ward-level inequality, we used the income inequality subdomain from the Indices of Deprivation to (v) estimate a Gini-like coefficient of the variance (i.e. inequality) in income deprivation scores within each ward at LSOA level, using the LSOA-ward weighting methodology described above (see also (9)). Finally, at regional-level (unavailable at ward-level), we obtained (vi) sex-specific, age-standardised prevalence of self-reported cannabis use in the population in the past year, estimated from the Adult Psychiatric Morbidity Survey of England [APMS] 2014 (10) as the proportion of respondents who reported using cannabis in the past year. All variables were treated as continuous covariates in our models, with the exception of deprivation, which was based on percentiles and classified into 20 quantiles.

# Seed dataset: Denominator details

Denominator data on the populations at-risk in each catchment area were estimated from the nearest decennial Census of Great Britain (ÆSOP/ELFEP: 2001; SEPEA: 2011) and multiplied by the duration of case ascertainment to obtain person-years at risk, as previously detailed (1,2,11).

# Prior distribution specifications

Where information was available, we used informative priors to describe the state of science on the model’s parameters (i.e. predictor variables), guided by the previous literature. When little or no literature or prior knowledge was available, we specified vague, minimally informative prior distributions for the relevant parameters. While providing only limited information on the uncertainty underlying these parameters, this choice ensured reasonable restrictions on them. In both cases, because the size of our dataset was large, we would expect the empirical data to dominate the priors in the posterior distributions that can be used to fully characterise the uncertainty around plausible values of incidence rate ratios. In the case of cannabis use, the inclusion of informative priors allows us to model this predictor in the absence of empirical data from the seed model about the effect of cannabis use on the relative risk of psychosis.

We describe the sources of prior information and the form of these priors for all individual-level and area-level covariates included in our six Poisson regression models, as follows.

## Age, sex and age-sex interaction term

Priors for age, sex, and the age-sex interaction were derived from Hardoon et al. (12).  We estimated the overall rates of all psychotic disorders by age and sex by summing the reported rates schizophrenia, bipolar disorders and other psychoses (Supplemental Table 2, Supplemental Figure 1). The reference group was set to females aged 16-17 years. There was no further information comparing 17-18 or 20-24 to 16-17 year olds; therefore, we included a non-informative prior for these age groups using a normal distribution with zero mean and a variance of 4, on the log scale. This is considered reasonably vague given the small incidence rate of FEP. We estimated the interaction based on the prior for sex and the incidence rate ratio [IRR] comparing males to females (Supplemental Table 2). Full prior distributions by age group and sex are listed in Supplemental Table 3.

## Ethnicity

Priors for ethnicity were derived from a meta-analysis by Bourque et al. (13) (Supplemental Table 4), which estimated risk for psychotic disorders among first- and second-generation immigrants. Based on these data, the prior distributions (on a log scale) were estimated for black Caribbean, black African, Indian, Pakistani and Bangladeshi groups in our dataset. For example, these implied that, on average, the relative risk of developing psychotic disorders for people of black Caribbean ethnicity was 3.9 times greater than for the white population.

## Cannabis use prevalence

The prior we included for the prevalence of cannabis use was derived from Moore et al. (14), which showed an increased risk of any psychotic outcome in individuals who had ever used cannabis (pooled adjusted odds ratio [OR]=1.41, 95% CI: 1.20 to 1.65). Using this odds ratio, we estimated the incremental effect of cannabis use in a given area (i.e. Census merged ward). At a population-level, we interpreted this odds ratio as corresponding to the (hypothetical) increase in the incidence of psychotic disorders which would occur in a given area if 100% of the population had smoked cannabis in the previous year. As a result, the prior distribution was specified as a normal distribution with a mean of 0.344 and a variance of 0.01 a log scale (Supplemental Figure 2). This was applied to the sex-specific values of self-reported cannabis use in the adult population in each region of England from the Adult Psychiatric Morbidity Survey (10) (Supplemental Table 5).

## Socioenvironmental variables

Typically, data such as the index of multiple deprivation [IMD] are modelled assuming a linear trend, which is often an unrealistic assumption (2). To overcome this issue, we chose to model IMD percentiles using a random walk prior. This amounts to allowing a flexible relationship between the outcome and equally-spaced intervals across IMD percentiles, based on the assumption that areas with similar deprivation scores were more likely to have similar FEP rates (see, for example, Supplemental Figure 3). The random walk prior allows the model to adapt the form of this relationship depending on the signal provided by the data, thus enabling a more precise estimation of the (posterior) effect of this potentially important predictor.

We were unable to identify suitable data that could be used as an informative prior for social fragmentation and population density, therefore we included non-informative priors for these variables. We set the variance of these non-informative prior distributions to be reasonably small (equal to 0.25 or 4 on the log scale) because the incidence of psychotic disorders is not very large (15).

# Projected denominator data for England up to 2025

We used a method developed by Rees et al. (16) to estimate the projected population at-risk in each Census merged ward up to 2025. Population projections up to 2025 were modelled on lower-tier local authority [LA] level using 2011 census data as a baseline, accounting for LA-level year-on-year changes in birth and death rates, immigration and emigration flows by ethnicity, and including assumptions about the impact of Brexit beyond 2020. The LA level data were disaggregated pro-rata to ward level, keeping the 2011 census proportions constant over time. We adopted a “hard” Brexit scenario, assuming that over time net migration would reach 100,000, consistent with the ruling Conservative Party’s target to reduce net international migration to “tens of thousands” as part of their 2010 General Election campaign pledge. This was followed by a decade of assertive immigration restrictions imposed by the Home Office’s immigration policy, which became colloquially referred to as the “hostile environment policy”. For the purpose of our forecasting, we assumed that after Brexit, immigration patterns for EU nationals would follow have the same pattern as observed for people who already required entry visas to live and work in the UK. We assumed that the combined impact of these policies, including the Brexit referendum in 2016 and formal exit from the EU in 2020, would begin to impact on net migration from 2014/15. We assumed the long-term limit of net migration at 100,000 per year would be achieved by 2031-32.

# FEP prediction: Further details

Model predictions were aggregated from Census Merged Ward level to Clinical Commissioning Group boundaries. To do so, we multiplied the matrix of posterior regression coefficients generated with the matrix, which contained census data at Census merged ward level. This process produced a matrix, which contained 1000 simulations for each ward stratified by age group, sex and ethnicity, allowing us to aggregate predictions for any desired combination of strata (e.g. totals, men, women, by ethnic group, by age group), at any desired geographical aggregation of ward level data for a given year. Simulations from relevant wards within a CCG were then aggregated, e.g. this gives 1000 simulations for each CCG (also stratified by these variables). We then summarised the mean and 95% intervals from these simulations to get final predictions at CCG level.

For validation against the 2017-18 MHSDS observed data, we used 2017 CCG boundaries. For prediction forecasting of our validated model between 2019 and 2025 we used the latest available CCG boundaries (2019) at the time of publication. A handful of 2011 census merged ward boundaries were not contiguous with CCG boundaries (i.e. 61 wards (0.79%) were split across 2 CCGs in 2017 and 1 was split across 3 CCGs (0.01%). For these 62 wards, we reweighted aggregated FEP prediction data at CCG-level based on the proportion of a ward’s total area belonging to each CCG. Of 7,678 wards, 99.19% fell within the catchment area of a single CCG in 2017. For 2019 CCG boundaries, corresponding values were: 99.49% of wards fell within the catchment area of a single CCG, with 78 (1.01%) split across 2 CCGs.

Prediction data were aggregated to and stratified by broad age group (16-64, 16-35 and 36-64 years old). Since narrower stratum-specific age groups used in our prediction models (i.e. “30-34”, “35-39”) were not coterminous with the 35/36 year old transition, we reweighted FEP predictions for any “35-39” year old strata when aggregated for either the 16-35 or 36-64 year old predictions. To do so, we derived the proportion of people aged 35 or 36-39 as a percentage of the 35-39 year old population in each Census merged ward, according to single-year-of-age data published in the 2011 Census (Census Table DC1117EW). These proportions were then applied to our prediction data to estimate the number of predicted cases aged 35 assigned to the 16-35 year-old age group, and the number of 36-39 year olds assigned to the 36-64 year old group, from the 35-39 year old prediction strata.

# Model validation: Further details of observed caseload definitions

Predicted FEP cases from our models were compared against observed data on *probable FEP* cases from the 2017-2018 financial year of reporting in NHS Digital’s Mental Health Services Dataset [MHSDS]. To define *probable FEP*, we derived four nested prediction types from the observed data, defined as follows:

**Referred to mental health services for “suspected psychosis” [referred caseload]:** All people referred to mental health services (not solely the EIP care pathway) where the primary referral reason recorded in the MHSDS was “suspected psychosis”.

**Accepted for assessment by EIP services [assessed caseload]:** A subset of the referred caseload who did not have a “referral rejection date” recorded in the MHSDS.

**Commenced EIP treatment for FEP [treated caseload]:** A subset of the assessed caseload with a recorded “clock-stop date” in the MHSDS. A “clock-stop date” is recorded as part of compliance to one of the NICE Access and Waiting Time Standards for EIP services (17), which mandates that 50% of all referrals accepted onto EIP caseloads must commence a “NICE-concordant package of care” within 14 days of the initial referral date. A clock-stop date denotes the date on which this treatment had commenced, meaning at least one attended contact with a care-coordinator.

**Probable FEP cases [probable cases]:** Because of the way referrals to and through EIP care are recorded in the MHSDS, not all people who received a “clock-stop date” are necessarily people with a clinically-validated diagnosis of ICD-10 psychotic disorder. Clock-stop dates may be assigned to clients who are alternatively defined as an At-Risk Mental State [ARMS], usually by fulfilling Comprehensive Assessment of At-Risk Mental States [CA-ARMS] criteria for psychosis vulnerability short of threshold criteria for psychotic disorder (18). They may also be assigned to people presenting with sufficient psychotic symptoms to warrant EIP treatment, but whose symptoms do not proceed to meet diagnostic criteria for psychotic disorder over the care period. Because symptom and diagnostic data are not routinely captured by the MHSDS, we had to use empirical evidence from EIP services to estimate the “false positive” rate of treated FEP cases likely to be captured within the MHSDS data. Previous research has suggested this is approximately 14% of those accepted for treatment onto EIP caseloads (2). We therefore derived *probable FEP* cases as an 86% subset of the treated caseload for comparison against our predicted FEP cases.

All prediction types (*referred/assessed/treated/probable*) were based on the assumption of being an incidence case. Participants were tracked back through previous iterations of the NHS Digital MHSDS, since 2013. This database was known as the Mental Health Minimum Dataset [MHMDS] between 2013-14, and the Mental Health and Learning Disabilities Dataset [MHLDS] between 2014-2016. Participants with a previous contact for suspected psychosis were excluded from the observed incidence data in the 2017-18 reporting period.

In the 2017-18 reporting period, EIP services in England operated across different age ranges for EIP treatment. To account for this during validation, we identified the age ranges served by EIP providers across CCGs, as reported in the 2017/18 EIPN self-assessment audit.(19) Of the 205 CCG included in the validation analysis, 87.8% (n=180) provided services to children and young people (<18 years), 88.3% (n=181) of CCGs provided services to persons over 35 years of age, and 81.5% (n=167) provided services to the full 16-64 age range. We matched predicted and observed caseloads according to these age ranges for validation.

# Sensitivity analyses to impute missing observed probable FEP data on ethnicity during external model validation against MHSDS data, 2017

In the MHSDS data, 820 observed *probable FEP* cases were missing data on ethnicity, which could have influenced ethnicity-specific model validation without correction. To examine this possible effect, we considered three alternative imputation scenarios in sensitivity analyses (see Supplemental Table 9 for results). In each scenario, we allocated the observed cases missing ethnicity to different ethnic groups, and re-estimated the difference between predicted and (adjusted) observed caseloads. These sensitivity analyses suggested that allocating cases proportionally across ethnic minority groups (Scenario #2, Supplemental Table 9) minimised error differences in all ethnic groups, except Bangladeshi (minimised in Scenario #3) and “other” ethnic groups (minimised in Scenario #1).

## Scenario #1: All missing values are assigned to the white British, Irish and Traveller

*Imputation method:* We assigned observed *probable FEP* cases with missing ethnicity to the “white British, Irish, and Traveller” category (i.e. the baseline, reference group).

*Rationale:* While reasons for missing ethnicity data are unknown, it is plausible that ethnicity may not be recorded routinely in clinical care where a participant’s ethnicity was assumed to fall in the majority “white British, Irish, and Traveller” category by the clinician responsible for making this assessment.

*Result:* Under this scenario, the differences between observed and predicted *probable FEP* caseloads became considerably larger in the “white British, Irish, and Traveller” category compared with the original complete case scenario (i.e. error differences between predicted and observed caseloads, expressed as a proportion of the (adjusted) observed caseload, changed from -2.4% to -17.0%; Supplemental Table 9), but errors in the ethnic minority categories were unaffected and remained high (varying from -33.4% in the “other” ethnic category to +96.4% in the “mixed” ethnic category).

## Scenario #2: All missing values are assigned to ethnic minority categories proportionally to the distribution of predicted FEP cases across all ethnic minority groups

*Imputation method:* We assigned observed *probable FEP* cases with missing ethnicity to an ethnic minority category in proportion to the distribution of FEP cases from the predicted model by ethnicity.

*Rationale:* While reasons for missing ethnicity data are unknown, it is plausible that ethnicity may not be recorded routinely in clinical care, if a participant is from an ethnic minority background, but their exact ethnicity could not be ascertained at the time of assessment.

*Results:* Under this scenario, we observed no change in the error difference between predicted and observed *probable FEP* caseloads, in the “white British, Irish, and Traveller” category (error difference: -2.4%). Differences for most ethnic minorities decreased and varied from -42.2% for the “other” ethnic category to +35.0% in the “mixed” ethnic category).

## Scenario #3: Missing values are assigned proportionally to the distribution of predicted FEP cases across all ethnic groups

*Imputation method:* We assigned observed *probable FEP* cases with missing ethnicity to all ethnic groups in proportion to the distribution of FEP cases from the predicted model by ethnicity.

*Rationale:* While reasons for missing ethnicity data are unknown, it is plausible that this occurred non-differentially with respect to ethnicity, thus all missing cases were distributed according to the proportion of predicted *probable FEP* cases by ethnicity.

*Result:* Under this scenario, the differences between observed and predicted *probable FEP* caseloads became larger in the “white British, Irish, and Traveller” (i.e. error difference proportion changes from -2.4% to -11.2%; Supplemental Table 9). Errors in the ethnic minority categories become smaller than under the complete case scenario (varying from -37.5% in the “other” ethnic category to +64.0% in the “mixed” ethnic category).

Overall, we interpret Scenario #1 as providing the worst imputed fit between the observed and predicted *probable FEP* data by ethnicity. Scenario #3 reduces error in most ethnic minority categories, but increases error in the “white British, Irish and Traveller” category. Scenario #2 provides the optimal fit of the three imputation scenarios considered here. This scenario results in less error than the complete case approach reported in Table 3, and while percentage errors remain high for some groups (mixed, other, black African and Indian), absolute errors at the national level are small and in the range -278 (“other”) to +133 (black African), equivalent to a mean absolute under-prediction of 1.4 *probable FEP* cases per CCG in the “other” ethnic category per year, and a mean absolute over-prediction of 0.6 black African *probable FEP* cases per CCG per year.

# Prediction forecasting, 2019-2025: Forecasting various prediction types

In addition to the predicted *probable FEP* cases per annum we forecasted between 2019-2025, we also estimated forecasts of the total number of new *referred*, *assessed* and *treated* caseloads (see Supplemental Materials, section 7 for definitions) per annum in England over this period. To do so, we used the following methodology: first, we multiplied predicted *probable FEP* caseload values by 1.16 to yield the *treated caseload* per annum. This ratio is the reciprocal of the original adjustment made in Section 7, based on evidence that the observed *probable FEP* was 86% of the *treated* caseload sample in previous empirical evidence (2). Next, for each of the 205 CCGs used to validate our models, we derived the ratio of observed “referred:treated” and “assessed:treated” caseloads reported in the MHSDS during the 2017-18 financial year. From these, we estimated the median “referred:treated” (2.33:1) and “assessed:treated” (2.10:1) ratios in England and multiplied the predicted *treated* caseload by these values to yield the predicted new *referred* and *assessed* caseloads per annum, respectively. Thus, for any prediction category (total, by broad age group, sex, ethnicity) at both CCG and national levels, we were able to estimate annual predicted new *referred/assessed/treated/probable FEP* caseloads, along with uncertainty, expressed via corresponding 95% intervals. In reporting these prediction forecasts, we derived both counts and rates of incidence caseloads over these four prediction types. Rates were expressed per 100,000 person-years.

# Supplemental References

1. Kirkbride JB, Fearon P, Morgan C, Dazzan P, Morgan K, Tarrant J, et al. Heterogeneity in incidence rates of schizophrenia and other psychotic syndromes: findings from the 3-center AeSOP study. Arch Gen Psychiatry. 2006;63(3):250–8.

2. Kirkbride JB, Hameed Y, Ankireddypalli G, Ioannidis K, Crane CM, Nasir M, et al. The Epidemiology of First-Episode Psychosis in Early Intervention in Psychosis Services: Findings From the Social Epidemiology of Psychoses in East Anglia [SEPEA] Study. Am J Psychiatry. 2017;174(2):143–53.

3. Kirkbride JB, Barker D, Cowden F, Stamps R, Yang M, Jones PB, et al. Psychoses, ethnicity and socio-economic status. Br J Psychiatry. 2008;193(1):18–24.

4. Wing JK, Babor T, Brugha T, Burke J, Cooper JE, Giel R, et al. Schedules for clinical assessment in neuropsychiatry. Arch Gen Psychiatry. 1990;47(6):589–93.

5. McGuffin P, Farmer A, Harvey I. A polydiagnostic application of operational criteria in studies of psychotic illness: Development and reliability of the OPCRIT system. Arch Gen Psychiatry. 1991;

6. Congdon P. Suicide and parasuicide in London: A small-area study. Congdon P, editor. Urban Stud. 1996;33(1):137–58.

7. Noble M, Wright G, Dibben C, Smith GAN, McLennan D, Anttila C, et al. Indices of Deprivation 2004. 2004.

8. Smith T, Noble M, Noble S, Wright G, McLennan D, Plunkett E. The English Index of Multiple Deprivation (IMD) 2015 - Technical Report. 2015.

9. Kirkbride JB, Jones PB, Ullrich S, Coid JW. Social deprivation, inequality, and the neighborhood-level incidence of psychotic syndromes in East London. Schizophr Bull [Internet]. 2012/12/14. 2014;40(1):169–80. Available from: https://www.ncbi.nlm.nih.gov/pubmed/23236081

10. Mcmanus S, Bebbington P, Jenkins R, Brugha T. Mental Health and Wellbeing in England: Adult Psychiatric Morbidity Survey 2014 Executive Summary [Internet]. Apms 2014. Leeds; 2016. p. 1–405. Available from: http://content.digital.nhs.uk/catalogue/PUB21748/apms‐2014‐exec‐summary.pdf.

11. Coid JW, Kirkbride JB, Barker D, Cowden F, Stamps R, Yang M, et al. Raised incidence rates of all psychoses among migrant groups: findings from the East London first episode psychosis study. Arch Gen Psychiatry. 2008;65(11):1250–8.

12. Hardoon S, Hayes JF, Blackburn R, Petersen I, Walters K, Nazareth I, et al. Recording of Severe Mental Illness in United Kingdom Primary Care, 2000–2010. PLoS One. 2013;8(12):e82365.

13. Bourque F, Van Der Ven E, Malla A. A meta-analysis of the risk for psychotic disorders among first- and second-generation immigrants. Psychol Med. 2010/07/29. 2011;41(5):897–910.

14. Moore THM, Zammit S, Lingford-Hughes A, Barnes TRE, Jones PB, Burke M, et al. Cannabis use and risk of psychotic or affective mental health outcomes: a systematic review. Lancet [Internet]. 2007;370(9584):319–28. Available from: https://pdf.sciencedirectassets.com/271074/1-s2.0-S0140673607X6032X/1-s2.0-S0140673607611623/main.pdf?X-Amz-Security-Token=AgoJb3JpZ2luX2VjEFIaCXVzLWVhc3QtMSJIMEYCIQC02wjegiROrVgQ0rezOicCiuTogCxcjZL6mDJAS%2F2SxQIhAIVZYwdbhdvZFsR%2Br6ekF1Bt7PRfU15iG6f7auO2

15. Kirkbride JB, Errazuriz A, Croudace TJ, Morgan C, Jackson D, Boydell J, et al. Incidence of schizophrenia and other psychoses in England, 1950-2009: a systematic review and meta-analyses. PLoS One. 2012;7(3):e31660.

16. Rees PH, Wohland P, Norman P, Lomax N, Clark SD. Population Projections by Ethnicity: Challenges and Solutions for the United Kingdom. In: Swanson D, editor. The Frontiers of Applied Demography Series 9. Springer, Cham; 2017. p. 383–408.

17. NHS England. Implementing the Early Intervention in Psychosis Access and Waiting Time Standard: Guidance. [Internet]. 2016. Available from: https://www.england.nhs.uk/mentalhealth/wp‐content/uploads/sites/29/2016/04/eipguidance.

18. Yung AR, Yuen HP, McGorry PD, Phillips LJ, Kelly D, Dell’Olio M, et al. Mapping the onset of psychosis: The Comprehensive Assessment of At-Risk Mental States. Aust N Z J Psychiatry. 2005;39(11–12):964–71.

19. Early Intervention in Psychosis Network, College Centre for Quality Improvement. Self-assessment for EIP Services 2017/18.

Supplemental Box 1. TRIPOD Checklist

| **Section/Topic** | **Item** |  | **Checklist Item** | **Page** |
| --- | --- | --- | --- | --- |
| **Title and abstract** | | | | |
| Title | 1 | D;V | Identify the study as developing and/or validating a multivariable prediction model, the target population, and the outcome to be predicted.  D;V: “Forecasting population need for early treatment programs in mental health using epidemiologic evidence: a generalisable Bayesian prediction methodology applied and validated for first episode psychoses in England” | 1 |
| Abstract | 2 | D;V | Provide a summary of objectives, study design, setting, participants, sample size, predictors, outcome, statistical analysis, results, and conclusions.  D;V – See Abstract. | 2 |
| **Introduction** | | | | |
| Background and objectives | 3a | D;V | Explain the medical context (including whether diagnostic or prognostic) and rationale for developing or validating the multivariable prediction model, including references to existing models.  D;V - Page 5: “Our methodology forecasted small area need for EIP provision up to 2025 to inform the NHS Long Term Plan (28) and Mental Health Implementation Plan in England (29)”  D;V - Page 4: “One example, previously applied to population-level need for EIP care in England, is known as the PsyMaptic model…” | 5 |
|  | 3b | D;V | Specify the objectives, including whether the study describes the development or validation of the model or both.  D;V - Page 5: “we developed a new, population-based prediction model applied to psychosis care in England, validated against observed national routine data” | 5 |
| **Methods** | | | | |
| Source of data | 4a | D;V | Describe the study design or source of data (e.g., randomized trial, cohort, or registry data), separately for the development and validation data sets, if applicable.  D - Pages 5-6: “Empirical incidence data were pooled from the three largest epidemiological catchment area studies…”  V - Pages 9-10: “we compared predicted FEP data, based on the 2017 denominator population, with observed national data available from NHS Digital’s Mental Health Services Dataset [MHSDS] in the closest (financial) year available (April 2017 – March 2018; henceforth, “2017”) … From this dataset, we extracted…”  Supp. pages 6-7:” To define probable FEP, we derived four nested prediction types from the observed data…” | D: 5-6;  V: 9-10, Supp. 6-7 |
|  | 4b | D;V | Specify the key study dates, including start of accrual; end of accrual; and, if applicable, end of follow-up.  D - Supp. page 3: “ÆSOP identified new cases of FEP in persons aged 16-64 years in Bristol (1997) over 9 months, and Southeast London and Nottingham over 2 years (1997-1999). ELFEP identified new cases of FEP in persons aged 18-64 in Newham (1996-1998), and Tower Hamlets and Hackney (1998-2000) over 2 years.”  V - See 4a. Also, Supp. page 6: “Participants were tracked back through previous iterations of the NHS Digital MHSDS, since 2013.” | D: S3;  V: 9-10, Supp. 6-7 |
| Participants | 5a | D;V | Specify key elements of the study setting (e.g., primary care, secondary care, general population) including number and location of centres.  D - Page 3:”Both studies ascertained cases from all potential secondary mental healthcare services in the relevant catchment areas, including private care. SEPEA identified new FEP cases aged 16-35 years old referred to EIP services in East Anglia over 3.5 years (2009-2013). For further details, see Kirkbride et al. (1–3).”  V - See 4a. | D: 3;  V: 9-10,  Supp. 6-7 |
|  | 5b | D;V | Describe eligibility criteria for participants.  D - Supp. page 3: FEP cases were defined as people who met standardised research criteria (ÆSOP/ELFEP: Schedule for Clinical Assessments in Neuropsychiatry [SCAN](4); SEPEA: Operationalised Criteria for Psychotic Illness [OPCRIT] (5)) for a diagnosis of International Classification of Disease, tenth revision [ICD-10] non-affective psychotic disorder (F20-29), affective psychotic disorder (F30-33) or substance-induced psychotic disorder (F1X.5). Individuals with an organic basis to their disorder, or profound learning difficulty were excluded.”  V - See 4a. | D: S3;  V: 9-10, Supp. 6-7 |
|  | 5c | D;V | Give details of treatments received, if relevant. | n/a |
| Outcome | 6a | D;V | Clearly define the outcome that is predicted by the prediction model, including how and when assessed.  D;V - See 4a (Supp. pages 6-7). | D;V: Supp. 6-7 |
|  | 6b | D;V | Report any actions to blind assessment of the outcome to be predicted. | n/a |
| Predictors | 7a | D;V | Clearly define all predictors used in developing the multivariable prediction model, including how and when they were measured.  D;V - Page 8: “We estimated the projected population-at-risk, aged 16-64, for all census merged wards in England (N=7,678) between 2017-2025, stratified by age group, sex, and ethnicity … For socioenvironmental covariates, we used the latest available routine data at the time of model development for deprivation (2015) (41),” social fragmentation (2011), population density (re-estimated for each year between 2017-2025, based on ward-level projected populations) and regional cannabis prevalence (2014) (42)”  Supp. page 3: “We used the following sources to define socioenvironmental variables in the seed dataset…”  Supp. pages 4-5 see Prior specification details | D;V: 8  Supp. 3-5 |
|  | 7b | D;V | Report any actions to blind assessment of predictors for the outcome and other predictors. | n/a |
| Sample size | 8 | D;V | Explain how the study size was arrived at. | n/a |
| Missing data | 9 | D;V | Describe how missing data were handled (e.g., complete-case analysis, single imputation, multiple imputation) with details of any imputation method.  D – n/a  V – Page 10: Ten (0.1%) observed probable FEP cases were missing information on sex, and excluded from sex-specific validation. Ethnicity data were missing for 820 (10.2%) observed probable FEP cases, which would have affected model validity by ethnicity without imputation, which we handled via imputation in sensitivity analyses (Supplemental Materials, section 8). | D: n/a  V: 10 |
| Statistical analysis methods | 10a | D | Describe how predictors were handled in the analyses.  D – See 7a. | D: 8, Supp. 3-5 |
|  | 10b | D | Specify type of model, all model-building procedures (including any predictor selection), and method for internal validation.  D – Page 7: “We modelled the count of new (incidence) cases of psychotic disorders using Poisson regression in a Bayesian framework … We fitted our models using Integrated Nested Laplace Approximation [INLA](37) to estimate the posterior distribution for the relevant parameters … Initial modelling suggested that ethnic density (RR=1.001; 95% interval, 0.998 to 1.005) and inequality (RR=0.90; 95% interval, 0.50 to 1.62) were not associated with FEP incidence in our seed dataset, and were removed from further consideration”.  page 9: “Apparent validity of the six candidate models was evaluated using the deviance information criterion [DIC], with a smaller value indicating better fit (33,44).” | 7-9 |
|  | 10c | V | For validation, describe how the predictions were calculated. | n/a |
|  | 10d | D;V | Specify all measures used to assess model performance and, if relevant, to compare multiple models.  D;V - See 10b (page 9). Also, page 10: “We inspected external validity at CCG-level by estimating correlation coefficients (r) between observed and predicted probable FEP counts, the r2-statistic, and root mean-squared error [RMSE], with higher r2 and lower RMSE indicating better model fit. We inspected calibration plots of predicted versus observed FEP counts and compared the difference in predicted and observed FEP counts, expressed as a rate per 100,000 person-years at CCG-level. At the national-level, we compared the difference between predicted and observed caseloads, expressed as a percentage, for the total count as well as by broad age group, sex and ethnic group. Choice of our best-fitting model was determined by consensus agreement between our authorship group after evaluation of all internal apparent and external validation metrics.” | 9-10 |
|  | 10e | V | Describe any model updating (e.g., recalibration) arising from the validation, if done. | n/a |
| Risk groups | 11 | D;V | Provide details on how risk groups were created, if done. | n/a |
| Development vs. validation | 12 | V | For validation, identify any differences from the development data in setting, eligibility criteria, outcome, and predictors. | n/a |
| **Results** | | | | |
| Participants | 13a | D;V | Describe the flow of participants through the study, including the number of participants with and without the outcome and, if applicable, a summary of the follow-up time. A diagram may be helpful.  D – See Kirkbride et al (2006, 2008, 2013).  V – Pages 14-15: “In 2017, 22,803 new referrals for “suspected psychosis” were recorded in the MHSDS across the 205 CCG included for validation; 89.9% (N=20,492) were subsequently accepted for EIP assessment, 35.2% (N=9,346) commenced EIP treatment and 35.2% (N=8,038; 86% of the treated caseload) met our definition of probable FEP.” | D: n/a  V: 14-15 |
|  | 13b | D;V | Describe the characteristics of the participants (basic demographics, clinical features, available predictors), including the number of participants with missing data for predictors and outcome.  D – See Table 1.  V – See Table 3. | D: Table 1  V: Table 3 |
|  | 13c | V | For validation, show a comparison with the development data of the distribution of important variables (demographics, predictors and outcome). | n/a |
| Model development | 14a | D | Specify the number of participants and outcome events in each analysis. | n/a |
|  | 14b | D | If done, report the unadjusted association between each candidate predictor and outcome. | n/a |
| Model specification | 15a | D | Present the full prediction model to allow predictions for individuals (i.e., all regression coefficients, and model intercept or baseline survival at a given time point).  D – See Supplemental Table 6. | Supplemental Table 6 |
|  | 15b | D | Explain how to use the prediction model.  D - Page 18. “Applied to FEP in England, our model provides healthcare commissioners with a decision-making tool to support the allocation of finite resources for EIP services, based on forecasts of need likely to arise in local populations, enhancing an earlier, more limited tool (18).” | 18 |
| Model performance | 16 | D;V | Report performance measures (with CIs) for the prediction model.  D; V - See Table 2. | Table 2 |
| Model-updating | 17 | V | If done, report the results from any model updating (i.e., model specification, model performance). | n/a |
| **Discussion** | | | | |
| Limitations | 18 | D;V | Discuss any limitations of the study (such as nonrepresentative sample, few events per predictor, missing data).  D;V – page 16-17: “Prediction models are influenced by their underlying assumptions and limitations…” | 16-17 |
| Interpretation | 19a | V | For validation, discuss the results with reference to performance in the development data, and any other validation data. | n/a |
|  | 19b | D;V | Give an overall interpretation of the results, considering objectives, limitations, results from similar studies, and other relevant evidence.  D;V – page 20: “Our best-fitting model yielded acceptable validity against observed routine data on probable FEP at CCG level in England for 2017”. | 16-20 |
| Implications | 20 | D;V | Discuss the potential clinical use of the model and implications for future research.  D;V – page 18: “Our predictions also allow care providers to understand the likely sociodemographic and environmental characteristics of their caseloads, which may be useful for providing locally and regionally sensitive modes of care delivery across diverse communities.”a | 16-20 |
| **Other information** | | | | |
| Supplementary information | 21 | D;V | Provide information about the availability of supplementary resources, such as study protocol, Web calculator, and data sets. | cited throughout |
| Funding | 22 | D;V | Give the source of funding and the role of the funders for the present study.  D;V - Page 21: See “Funding” section. | 21 |

*Items relevant only to the development of a prediction model are denoted by D, items relating solely to a validation of a prediction model are denoted by V, and items relating to both are denoted D;V. We recommend using the TRIPOD Checklist in conjunction with the TRIPOD Explanation and Elaboration document.

Supplemental Figure 1. Graphical representation of the prior distribution for the effect of male sex on psychosis risk in a single age group.


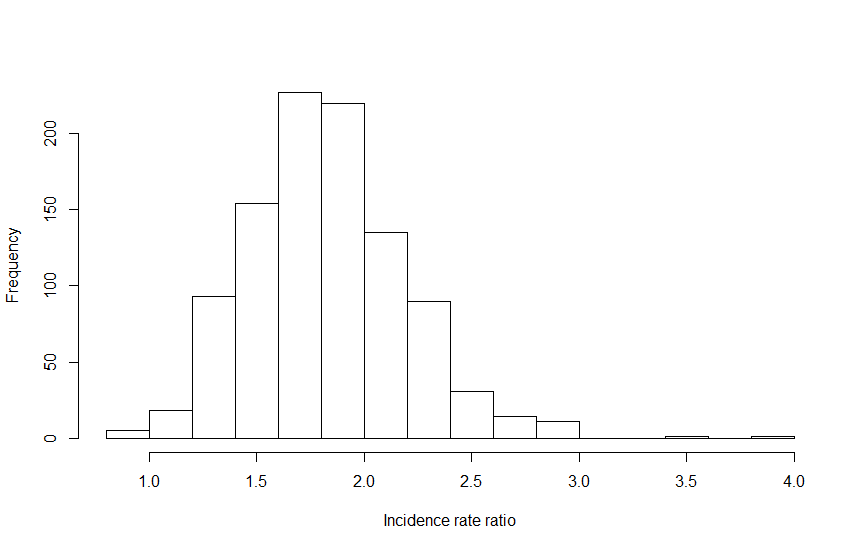


**Legend:** The prior distribution for male sex, which represents the IRR for males compared with females aged 16-17. The median RR is approximately 1.8 and there is 95% chance that this RR lies within a range of 1.4 to 2.4, which is reasonable given the small variance of both incidence rates (e.g. SD of 5) and RR (e.g. SD of 0.2). Since 16-17 year olds were the reference group, the prior here is solely for males versus females, as detailed in Supplemental Table 3. Similar prior distributions for other age groups were also estimated by combining the two relevant prior distributions (age specific prior with age-sex interaction term; Supplemental Table 3) to lead to a single combined distribution for each age group, allowing the prior distribution of the sex-specific IRR to vary by age group.


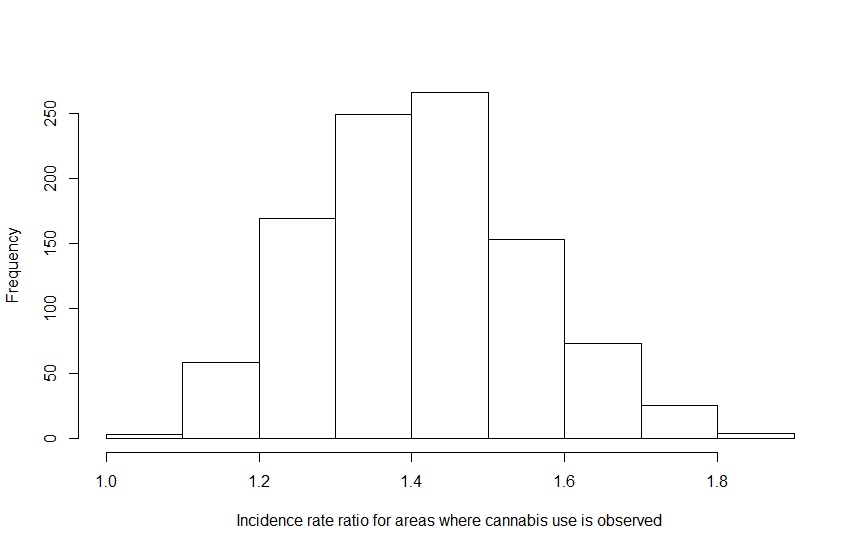
Supplemental Figure 2. Graphical representation of the prior distribution included for lifetime prevalence of cannabis use on psychosis risk.

**Legend**: Compared with a hypothetical area where no individual in the population at-risk had ever used cannabis, the incidence rate ratio for areas where everyone had used cannabis would centre around 1.4 (e.g. 40% higher rate of psychosis) with a 95% probability that the IRR lies within a range of 1.2-1.7, as reflected by the priori information provided by Moore et al (14).

Supplemental Figure 3. Plot showing the incidence rate per 100,000 by deprivation quantile among White British, Irish, and Traveller, females ages 16-17 years for Model 4.

**
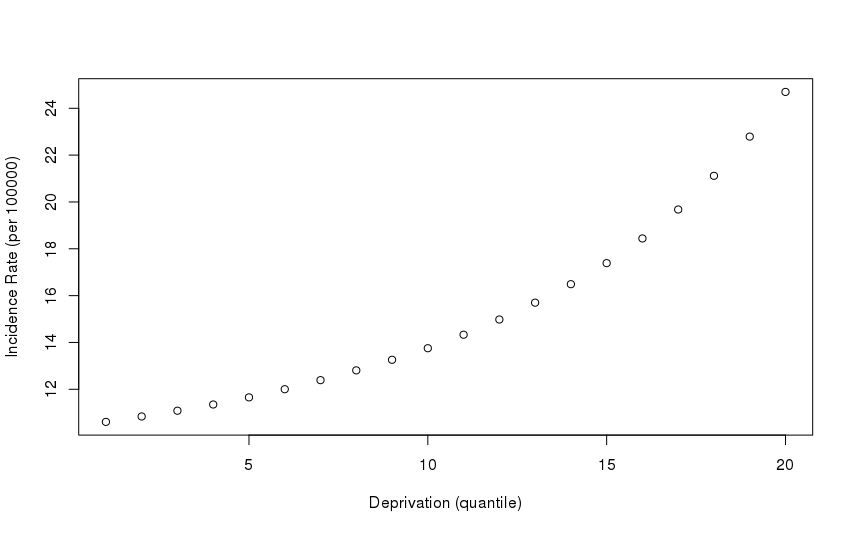
**

**Legend**: Based on Model 4, among White British, Irish, and Traveller females ages 16-17 years, the incidence rate of FEP increases in a non-linear fashion with increased area-level deprivation. Similar plots can be produced for each sociodemographic stratification. Plots were very similar for the other candidate models and show our prior distributional assumption about the expected relationship between FEP incidence and deprivation, fitted with a random-walk prior to allow for a non-linear association.

Supplemental Figure 4. Calibration plots of observed versus predicted counts of FEP cases in 205 CCGs for Models 1-6.


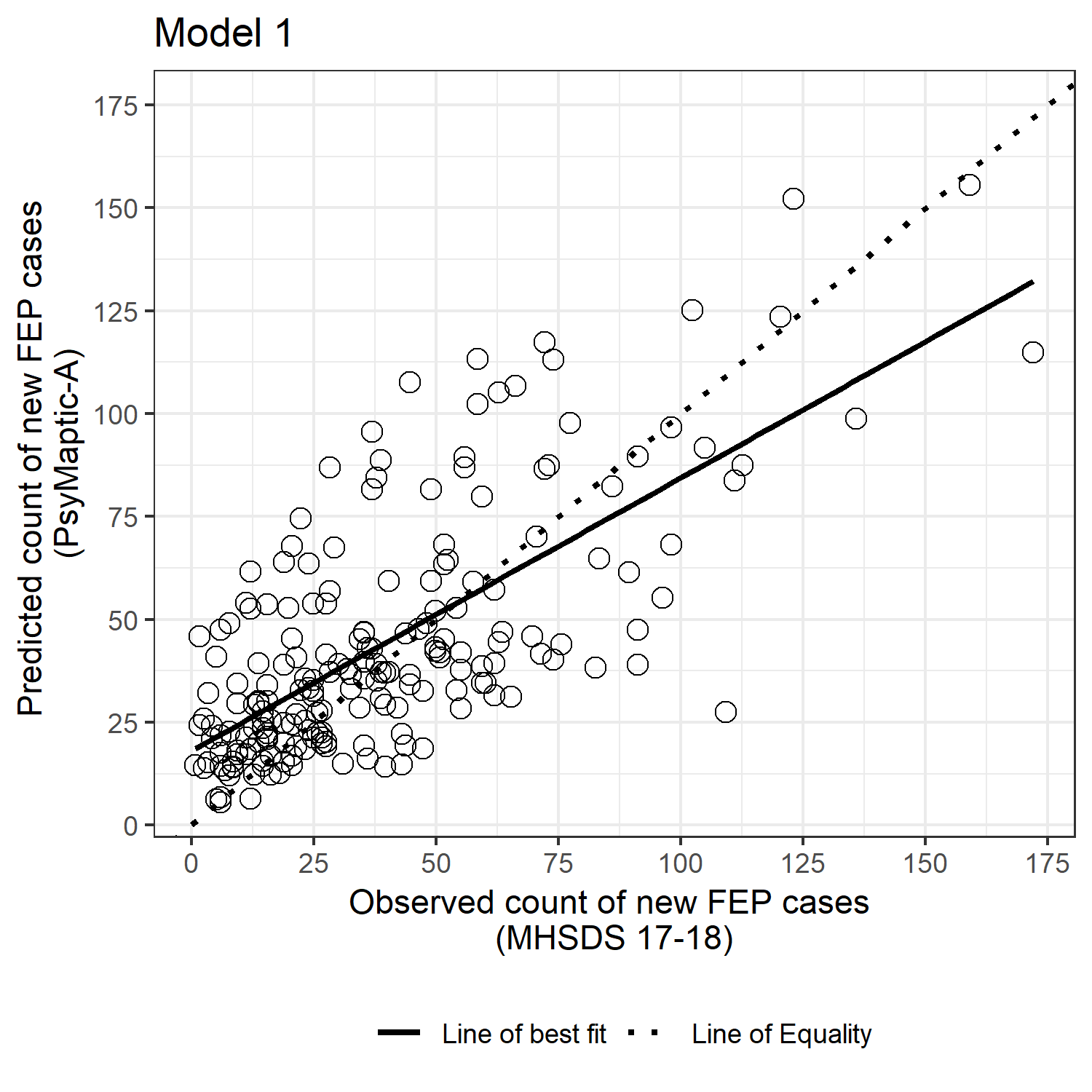

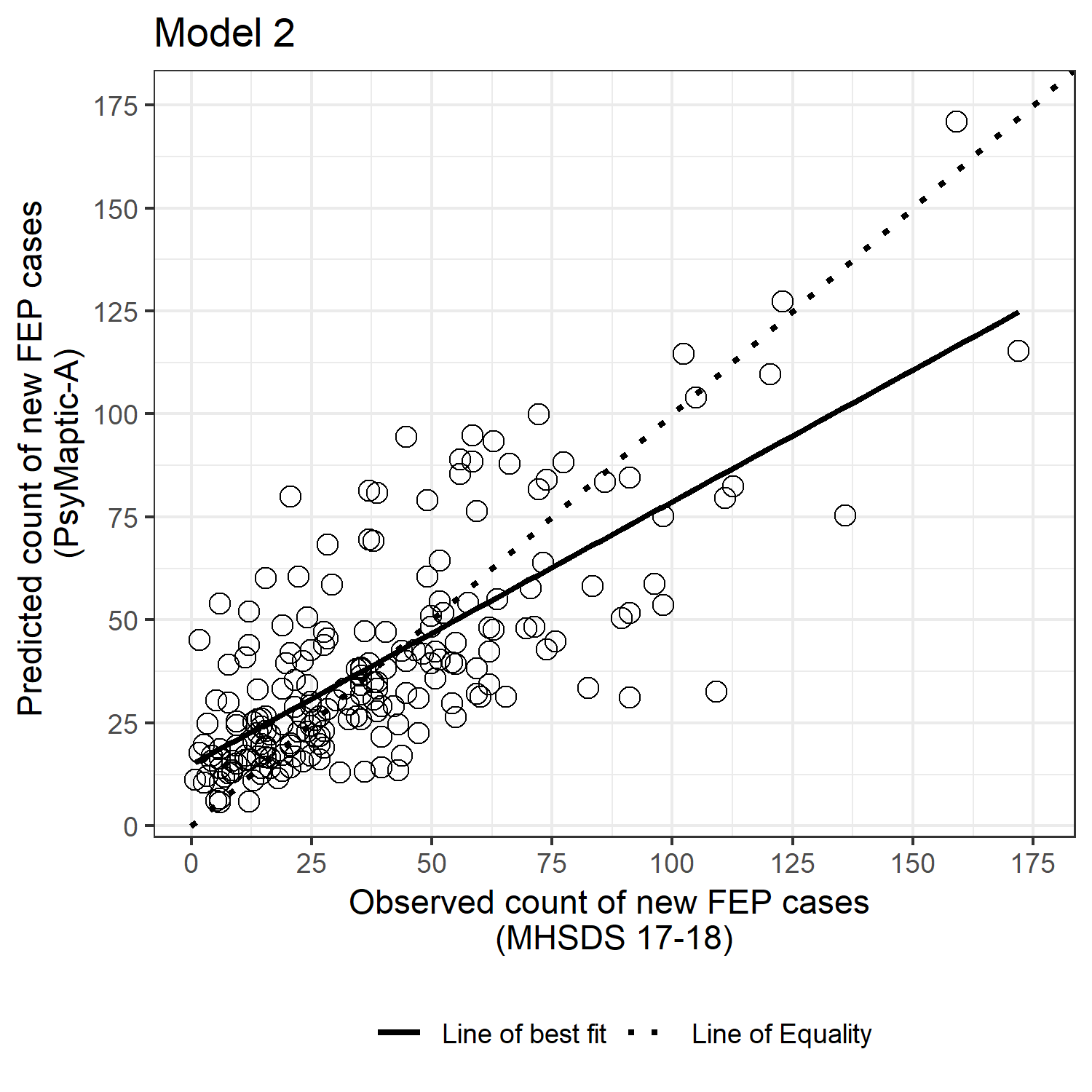

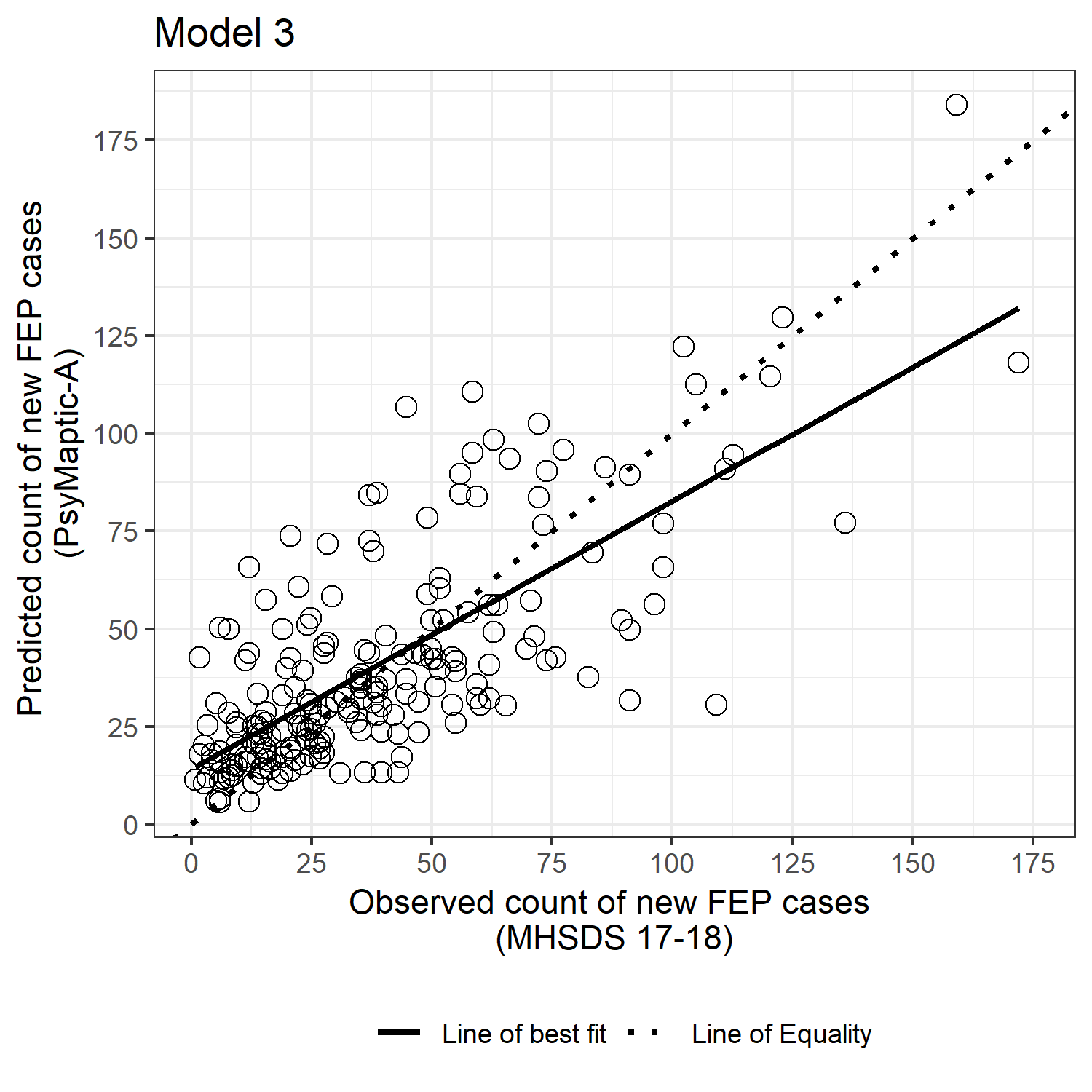

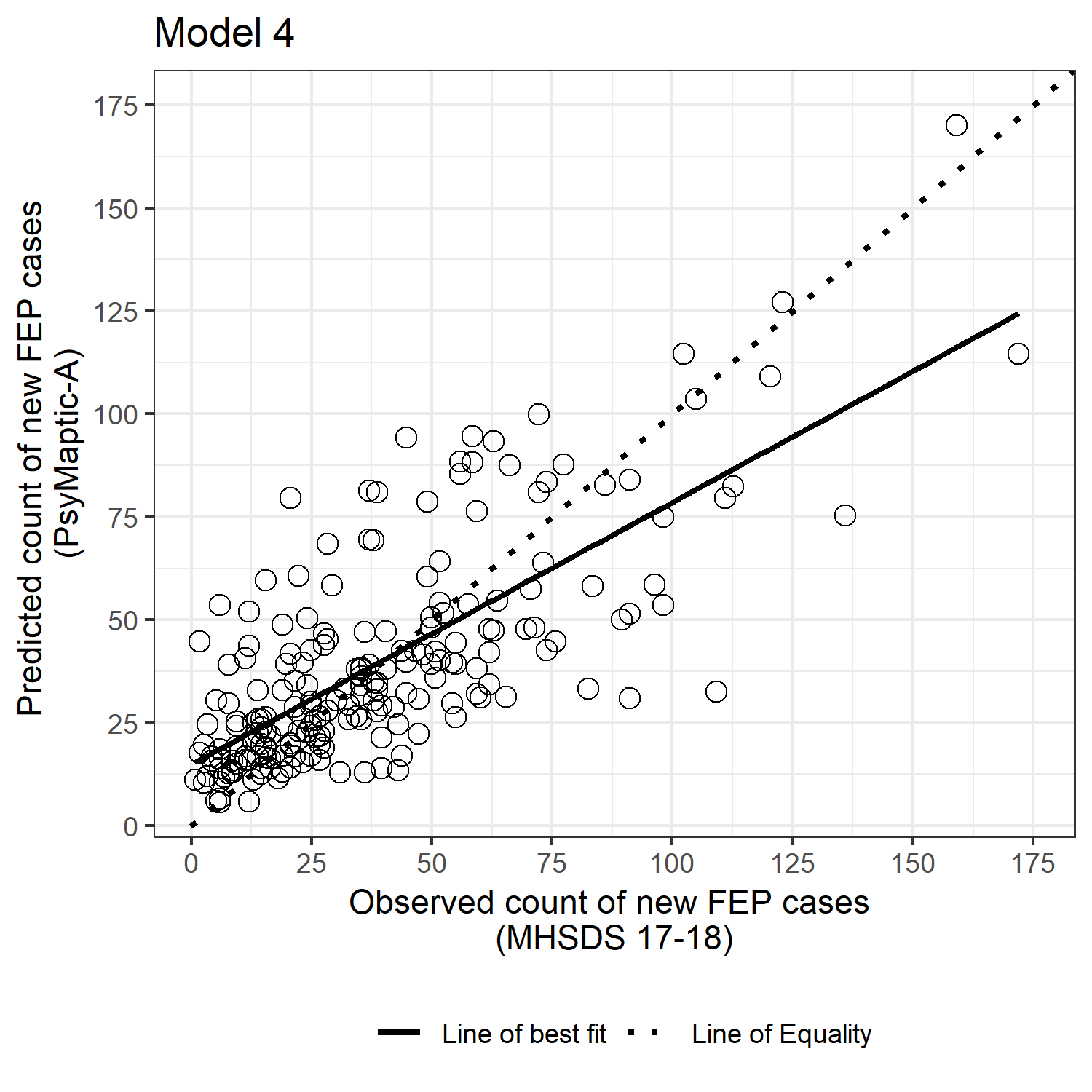

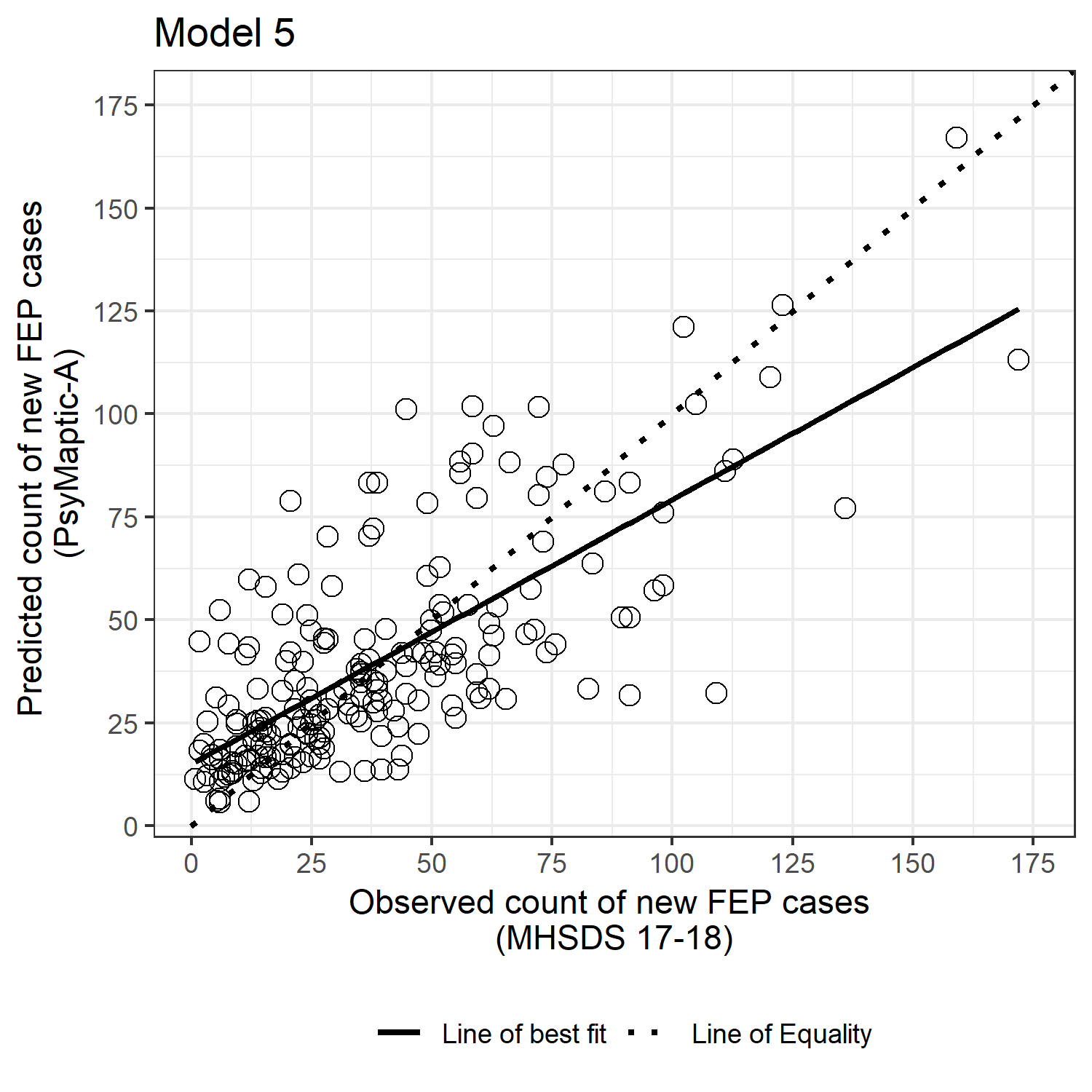

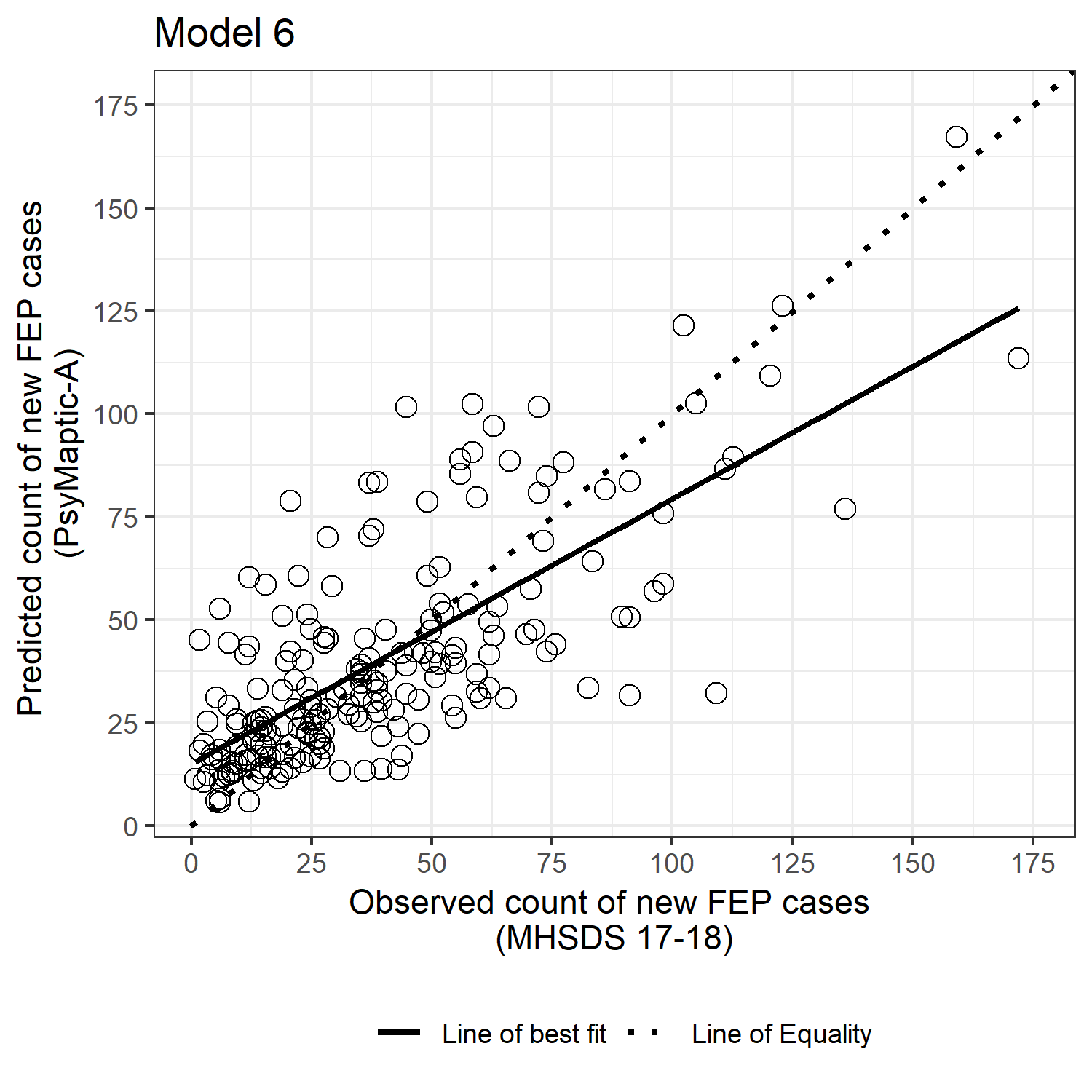


**Legend**: Observed and fitted values for the correlation between predicted FEP (y-axis) and observed *probable FEP* (x-axis) caseloads at CCG level (N=205) in England, 2017. The dashed line of equality indicates where observed and predicted caseloads would be the same; the solid line is the model-specific slope parameter having regressed observed caseloads on predicted caseloads (see Table 2 for calibration metrics). All slope parameters fell within the range 0.64 – 0.69, indicating a positive correlation between observed and predicted *probable FEP* caseloads at CCG level, although on average our models predicted between 64-69 FEP cases for every 100 observed; possible reasons for this are presented in the discussion section.

Figure 5. Visualisation of (A) predicted FEP caseloads, and (B) observed probable FEP caseloads, by CCG in England, 2017


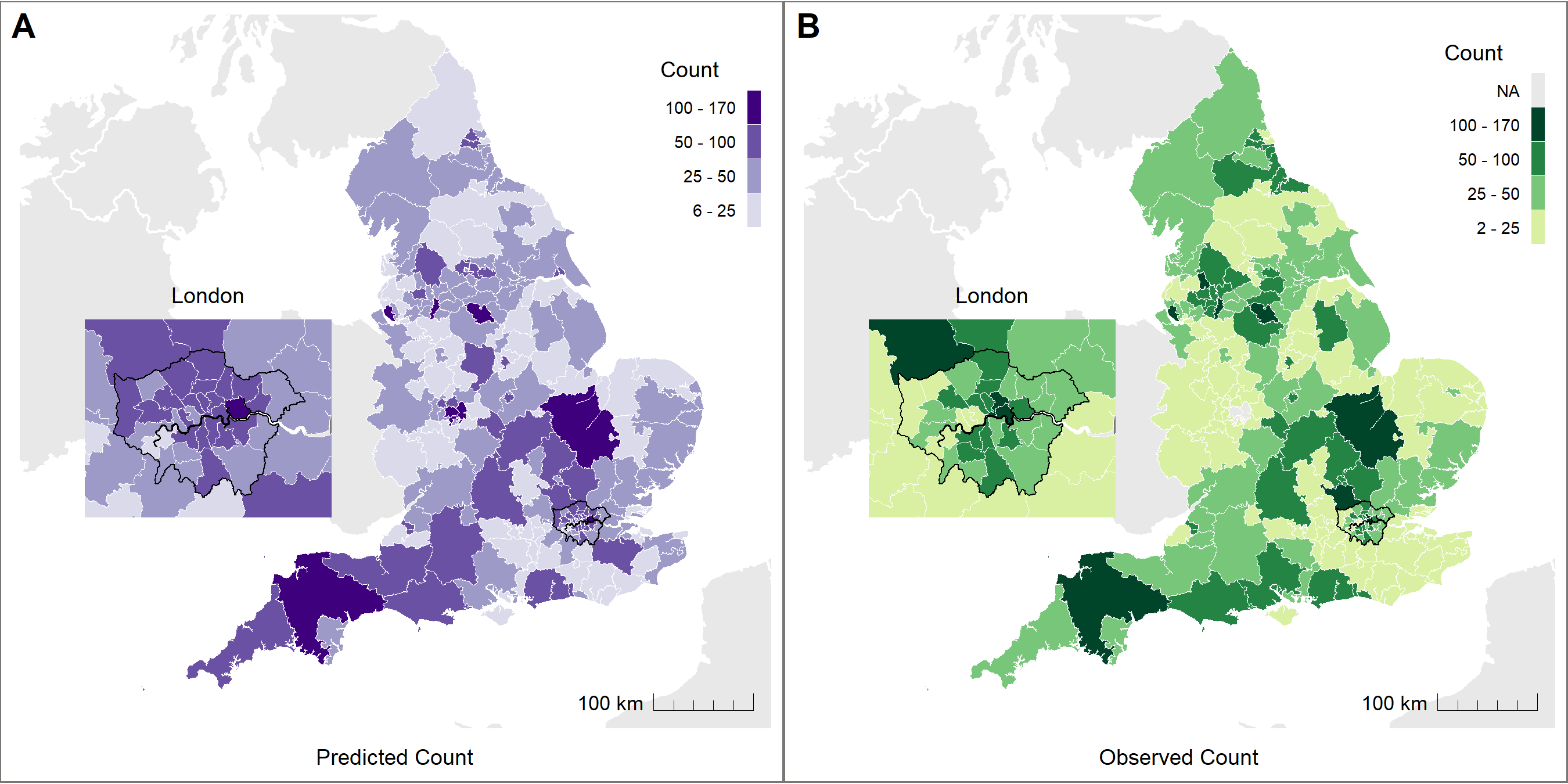


**Legend:** Observed FEP data from two CCG were excluded from validation due to data reliability issues (see methods); values are not shown in Panel B. Observed data were based on routine data from the NHS Mental Health Services Dataset [MHSDS] for the financial year April 2017 – March 2018

Supplemental Figure 6. Visualisations of (A) predicted rate of FEP per 100,000, (B) observed rate of probable FEP per 100,000, and (C) differences between predicted and observed caseload as a rate per 100,000 person-years, by CCG in England, 2017.


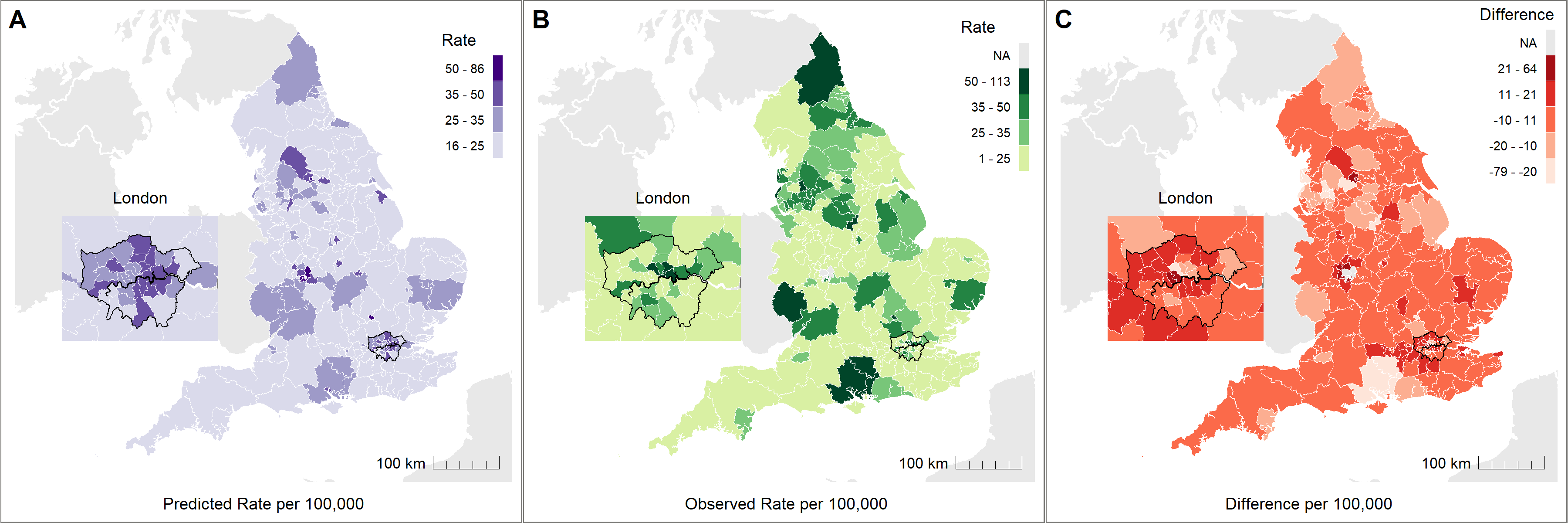


**Legend**: Predicted *probable FEP* rates (panel A) were matched to observed *probable FEP* rates by age ranges served by EIP services in each CCG (see methods). Observed FEP data from two CCG (see methods) were excluded from panels B & C due to data reliability issues.

Supplemental Figure 7. Predicted count of annual new referred, assessed, treated and probable FEP caseloads in England between 2019-2025, by broad age group.


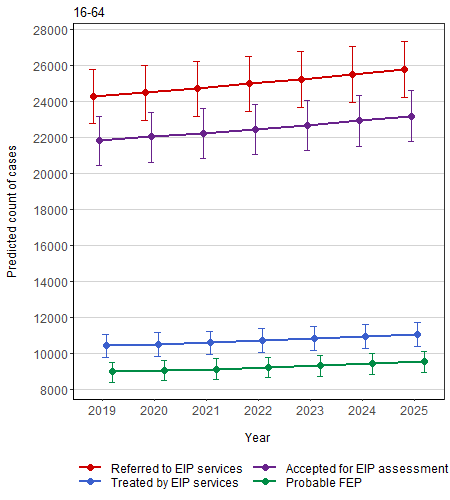

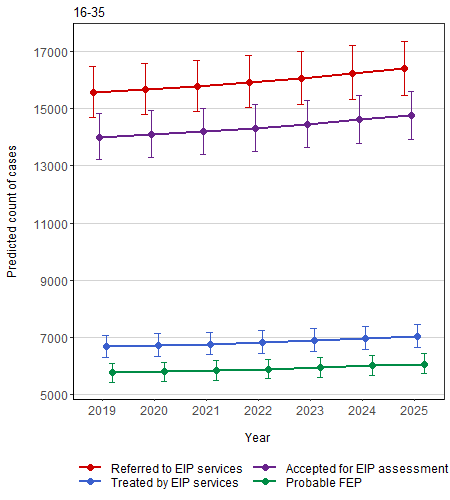

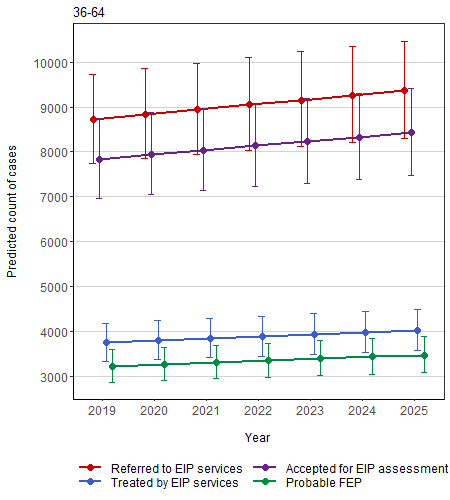


**A**

**B**

**C**

**Legend**: Predicted national count of caseload types, by broad age group in England between 2019-2025 for ages (**A**) 16-64 years; (**B**) 16-34 years, and (**C**) 35-64 years old. Annual average rises in caseloads increased 6.2% over the forecast period, but uncertainty around these estimates (see also Supplemental Table 10) meant we could not exclude the possibility of other trends in caseloads over time, including a possible decline or stable caseload sizes. Please note relative scale of caseload sizes on y-axis between panels.

Supplemental Table 1. Summary of distribution of cases across all strata of the seed data.^a^

|  | **Mean** | **Variance** |
| --- | --- | --- |
| **Total** | 0.03 | 0.03 |
| **Age Group** |  |  |
| 16-17 | 0.02 | 0.02 |
| 18-19 | 0.04 | 0.04 |
| 20-24 | 0.06 | 0.07 |
| 25-29 | 0.05 | 0.05 |
| 30-34 | 0.03 | 0.03 |
| 35-39 | 0.01 | 0.01 |
| 40-44 | 0.02 | 0.02 |
| 45-49 | 0.02 | 0.02 |
| 50-54 | 0.01 | 0.01 |
| 55-59 | 0.01 | 0.01 |
| 60-64 | 0.01 | 0.01 |
| **Sex** |  |  |
| Male | 0.04 | 0.04 |
| Female | 0.02 | 0.02 |
| **Ethnicity** |  |  |
| White British, Irish & Traveller | 0.07 | 0.08 |
| White Other | 0.02 | 0.02 |
| Black Caribbean | 0.04 | 0.04 |
| Black African | 0.04 | 0.03 |
| Indian | 0.01 | 0.01 |
| Pakistani | 0.01 | 0.01 |
| Bangladeshi | 0.02 | 0.02 |
| Mixed | 0.01 | 0.01 |
| Other | 0.01 | 0.01 |

^a^ Total informative strata = 54,432, comprised of 11 age groups, 2 sexes, 9 ethnic groups over 770 Census merged wards in the seed dataset with a non-zero denominator population at-risk. Of total strata (N=152,460), 98,028 strata contained no denominator (i.e. zero persons at-risk), and thus no useable information, and were dropped from the seed dataset.

Supplemental Table 2. Incidence rates of psychosis by age and sex derived from Hardoon et al. (12) used to derive prior distributions in Bayesian models.

| **Age group** | **Incidence rate^a^** | | **Ratio** | **Incidence rate ratio** | |
| --- | --- | --- | --- | --- | --- |
|  | **Male** | **Female** | **Male/Female** | **Male** | **Female** |
| 16-24 | 76.0 | 42.4 | 1.792 | 1 (ref.) | 1 (ref.) |
| 25-34 | 59.6 | 53.4 | 1.116 | 0.784 | 1.259 |
| 35-44 | 46.0 | 50.3 | 0.915 | 0.605 | 1.186 |
| 45-54 | 36.3 | 45.4 | 0.800 | 0.478 | 1.071 |
| 55-64 | 26.5 | 33.4 | 0.793 | 0.349 | 0.788 |

^a^ per 100,000 person-years at risk.

Supplemental Table 3. Prior distributions included in Bayesian models for each age-sex stratification, based on a normal distribution.

| **Age group** | **Female** | **Male** | **Interaction (effective)** | **Resulting distribution of IRR comparing M/F for a given age group** |
| --- | --- | --- | --- | --- |
| 16-17 | reference | N(0.583, 0.04) | reference | reference |
| 18-19 | N(0, 4) |  | N(0, 4) | N(0, 4)^a^ |
| 20-24 | N(0, 4) |  | N(0, 4) | N(0, 4)^a^ |
| 25-29 | N(0.23, 0.04) |  | N(-0.473, 0.04) | N(0.11, 0.04) |
| 30-34 | N(0.23, 0.04) |  | N(-0.473, 0.04) | N(0.11, 0.04) |
| 35-39 | N(0.171, 0.04) |  | N(-0.672, 0.04) | N(-0.089, 0.04) |
| 40-44 | N(0.171, 0.04) |  | N(-0.672, 0.04) | N(-0.089, 0.04) |
| 45-49 | N(0.069, 0.04) |  | N(-0.806, 0.04) | N(-0.223, 0.04) |
| 50-54 | N(0.069, 0.04) |  | N(-0.806, 0.04) | N(-0.223, 0.04) |
| 55-59 | N(-0.238, 0.04) |  | N(-0.815, 0.04) | N(-0.232, 0.04) |
| 60-64 | N(-0.238, 0.04) |  | N(-0.815, 0.04) | N(-0.232, 0.04) |

^a^ Vague (non-informative) priors were fitted given a lack of sufficient *a priori* data in these strata (see Supplemental Materials, section 4).

Supplemental Table 4. Incidence rates of psychosis by major ethnic group derived from Bourque et al. (13) used to derive prior distributions in Bayesian models.

| **Ethnicity** | **Incidence Rate Ratio (95% CI)** | | **Prior distribution** |
| --- | --- | --- | --- |
|  | **First generation** | **Second generation** | **Combined**^b^ |
| Black Caribbean | 3.9 (3.4 to 4.6) | 5.8 (3.5 to -)^a^ | Normal(1.36, 4) |
| Black African | 4.3 (2.8 to 6.8) | 3.7 (2.2 to 6.3) | Normal(1.31, 4) |
| Asian^c^ | 1.7 (1.3 to 2.3) | 1.3 (0.8 to 2.1) | Normal(0.26, 4) |

95% CI: 95% confidence interval.

^a^ Upper bound not reported.

^b^ Prior distribution for all generation groups combined, on natural logarithm scale. Mean and standard deviation.

^c^ Applied to priors for Indian, Pakistani and Bangladeshi groups in our prediction models.

Supplemental Table 5. Sex-specific prevalence of self-reported cannabis use in the adult population in each region of England, from the Adult Psychiatric Morbidity Survey 2014 (10).

|  | **Prevalence (%)^a^** | |
| --- | --- | --- |
| **Region** | **Female** | **Male** |
| North East | 4.5 | 9.0 |
| North West | 3.9 | 10.4 |
| Yorkshire & the Humber | 3.4 | 9.4 |
| East Midlands | 4.1 | 8.6 |
| West Midlands | 4.0 | 7.4 |
| East of England | 4.4 | 10.2 |
| London | 6.1 | 11.1 |
| South East | 6.3 | 7.2 |
| South West | 6.9 | 7.6 |

^a^ Reported in the previous year.

Supplemental Table 6. Posterior relative risks with corresponding 95% intervals for parameters from candidate models 1-6.

|  | **Model 1** | **Model 2^a^** | **Model 3^a^** | **Model 4^a^** | **Model 5^a^** | **Model 6^a^** |
| --- | --- | --- | --- | --- | --- | --- |
|  | **Age, sex, age*sex, ethnicity** | **1 + deprivation, social fragmentation** | **1 + deprivation, population density** | **2 + cannabis** | **4 + population density** | **2 + population density** |
|  | **RR (95% interval)** | **RR (95% interval)** | **RR (95% interval)** | **RR (95% interval)** | **RR (95% interval)** | **RR (95% interval)** |
| **Age Group** |  |  |  |  |  |  |
| 16-17 (ref.) | 1 | 1 | 1 | 1 | 1 | 1 |
| 18-19 | 1.86 (1.37 to 2.50) | 1.94 (1.43 to 2.61) | 1.93 (1.42 to 2.59) | 1.95 (1.43 to 2.61) | 1.96 (1.44 to 2.63) | 1.96 (1.44 to 2.63) |
| 20-24 | 1.61 (1.28 to 2.02) | 1.66 (1.31 to 2.08) | 1.63 (1.29 to 2.05) | 1.66 (1.31 to 2.08) | 1.67 (1.32 to 2.10) | 1.66 (1.32 to 2.09) |
| 25-29 | 1.46 (1.20 to 1.77) | 1.47 (1.21 to 1.79) | 1.46 (1.20 to 1.78) | 1.47 (1.21 to 1.79) | 1.48 (1.22 to 1.79) | 1.48 (1.21 to 1.79) |
| 30-34 | 1.04 (0.85 to 1.28) | 1.06 (0.87 to 1.30) | 1.06 (0.86 to 1.30) | 1.06 (0.87 to 1.30) | 1.06 (0.87 to 1.30) | 1.06 (0.87 to 1.30) |
| 35-39 | 1.04 (0.83 to 1.30) | 1.02 (0.81 to 1.27) | 1.01 (0.81 to 1.26) | 1.02 (0.81 to 1.27) | 1.01 (0.81 to 1.27) | 1.01 (0.81 to 1.27) |
| 40-44 | 0.99 (0.77 to 1.26) | 0.95 (0.74 to 1.22) | 0.94 (0.74 to 1.20) | 0.95 (0.74 to 1.21) | 0.95 (0.74 to 1.21) | 0.95 (0.74 to 1.21) |
| 45-49 | 0.95 (0.73 to 1.23) | 0.92 (0.70 to 1.19) | 0.91 (0.70 to 1.18) | 0.92 (0.70 to 1.19) | 0.91 (0.70 to 1.18) | 0.91 (0.70 to 1.18) |
| 50-54 | 0.86 (0.65 to 1.13) | 0.84 (0.63 to 1.10) | 0.83 (0.63 to 1.09) | 0.84 (0.63 to 1.10) | 0.83 (0.63 to 1.09) | 0.83 (0.63 to 1.09) |
| 55-59 | 0.68 (0.50 to 0.91) | 0.66 (0.49 to 0.88) | 0.66 (0.49 to 0.88) | 0.66 (0.49 to 0.88) | 0.66 (0.49 to 0.88) | 0.66 (0.49 to 0.88) |
| 60-64 | 0.58 (0.43 to 0.77) | 0.56 (0.41 to 0.75) | 0.56 (0.41 to 0.75) | 0.56 (0.41 to 0.75) | 0.56 (0.41 to 0.75) | 0.56 (0.41 to 0.75) |
| **Sex** |  |  |  |  |  |  |
| Male | 2.15 (1.78 to 2.59) | 2.17 (1.80 to 2.62) | 2.18 (1.80 to 2.63) | 2.14 (1.77 to 2.57) | 2.14 (1.77 to 2.58) | 2.18 (1.80 to 2.63) |
| **Age-Sex Interaction** |  |  |  |  |  |  |
| 16-17*Male (ref.) | 1 | 1 | 1 | 1 | 1 | 1 |
| 18-19*Male | 1.09 (0.77 to 1.56) | 1.09 (0.76 to 1.56) | 1.09 (0.76 to 1.56) | 1.09 (0.76 to 1.56) | 1.08 (0.76 to 1.55) | 1.08 (0.76 to 1.55) |
| 20-24*Male | 0.99 (0.75 to 1.30) | 0.99 (0.75 to 1.30) | 0.98 (0.75 to 1.29) | 0.99 (0.75 to 1.30) | 0.99 (0.75 to 1.30) | 0.99 (0.75 to 1.30) |
| 25-29*Male | 0.77 (0.61 to 0.96) | 0.77 (0.61 to 0.96) | 0.76 (0.61 to 0.96) | 0.77 (0.61 to 0.96) | 0.77 (0.61 to 0.96) | 0.77 (0.61 to 0.96) |
| 30-34*Male | 0.68 (0.53 to 0.86) | 0.68 (0.53 to 0.86) | 0.67 (0.53 to 0.86) | 0.68 (0.53 to 0.86) | 0.68 (0.53 to 0.86) | 0.68 (0.53 to 0.86) |
| 35-39*Male | 0.52 (0.39 to 0.68) | 0.51 (0.39 to 0.67) | 0.51 (0.39 to 0.66) | 0.51 (0.39 to 0.67) | 0.51 (0.39 to 0.67) | 0.51 (0.39 to 0.67) |
| 40-44*Male | 0.47 (0.35 to 0.64) | 0.47 (0.35 to 0.63) | 0.46 (0.34 to 0.62) | 0.47 (0.35 to 0.63) | 0.46 (0.34 to 0.62) | 0.46 (0.34 to 0.62) |
| 45-49*Male | 0.45 (0.33 to 0.61) | 0.44 (0.32 to 0.60) | 0.44 (0.32 to 0.60) | 0.44 (0.32 to 0.60) | 0.44 (0.32 to 0.60) | 0.44 (0.32 to 0.60) |
| 50-54*Male | 0.41 (0.29 to 0.57) | 0.40 (0.29 to 0.56) | 0.40 (0.29 to 0.56) | 0.40 (0.29 to 0.56) | 0.40 (0.29 to 0.56) | 0.40 (0.29 to 0.56) |
| 55-59*Male | 0.64 (0.46 to 0.89) | 0.63 (0.45 to 0.88) | 0.63 (0.45 to 0.88) | 0.63 (0.45 to 0.88) | 0.63 (0.45 to 0.88) | 0.63 (0.45 to 0.88) |
| 60-64*Male | 0.71 (0.51 to 0.99) | 0.70 (0.50 to 0.98) | 0.70 (0.50 to 0.97) | 0.70 (0.50 to 0.98) | 0.70 (0.50 to 0.97) | 0.70 (0.50 to 0.97) |

|  | **Model 1** | **Model 2^a^** | **Model 3^a^** | **Model 4^a^** | **Model 5^a^** | **Model 6^a^** |
| --- | --- | --- | --- | --- | --- | --- |
|  | **Age, sex, age*sex, ethnicity** | **1 + deprivation, social fragmentation** | **1 + deprivation, population density** | **2 + cannabis** | **4 + population density** | **2 + population density** |
|  | **RR (95% interval)** | **RR (95% interval)** | **RR (95% interval)** | **RR (95% interval)** | **RR (95% interval)** | **RR (95% interval)** |
| **Ethnicity** |  |  |  |  |  |  |
| White British, Irish and Traveller (ref.) | 1 | 1 | 1 | 1 | 1 | 1 |
| White Other | 1.59 (1.33 to 1.89) | 1.51 (1.26 to 1.80) | 1.47 (1.23 to 1.75) | 1.51 (1.26 to 1.80) | 1.50 (1.25 to 1.78) | 1.50 (1.25 to 1.78) |
| Black Caribbean | 5.69 (4.87 to 6.63) | 4.81 (4.09 to 5.64) | 4.61 (3.90 to 5.43) | 4.80 (4.08 to 5.63) | 4.64 (3.92 to 5.47) | 4.65 (3.93 to 5.47) |
| Black African | 4.01 (3.37 to 4.74) | 3.34 (2.79 to 3.98) | 3.21 (2.67 to 3.85) | 3.33 (2.78 to 3.97) | 3.20 (2.66 to 3.84) | 3.21 (2.67 to 3.84) |
| Indian | 1.38 (0.98 to 1.89) | 1.27 (0.90 to 1.75) | 1.23 (0.87 to 1.69) | 1.27 (0.90 to 1.75) | 1.23 (0.87 to 1.69) | 1.23 (0.87 to 1.69) |
| Pakistani | 2.25 (1.63 to 3.04) | 1.92 (1.38 to 2.61) | 1.87 (1.35 to 2.54) | 1.92 (1.38 to 2.60) | 1.88 (1.36 to 2.56) | 1.88 (1.36 to 2.56) |
| Bangladeshi | 2.35 (1.83 to 2.97) | 1.71 (1.32 to 2.19) | 1.65 (1.26 to 2.13) | 1.70 (1.31 to 2.18) | 1.61 (1.23 to 2.07) | 1.61 (1.23 to 2.08) |
| Mixed | 2.25 (1.72 to 2.89) | 2.11 (1.62 to 2.72) | 2.05 (1.57 to 2.64) | 2.11 (1.62 to 2.71) | 2.08 (1.59 to 2.68) | 2.09 (1.60 to 2.68) |
| Other | 1.48 (1.12 to 1.91) | 1.44 (1.09 to 1.87) | 1.37 (1.04 to 1.78) | 1.44 (1.09 to 1.86) | 1.41 (1.07 to 1.83) | 1.41 (1.07 to 1.83) |
| **Socioenvironmental Factors** |  |  |  |  |  |  |
| SFI (z-standardised) | - | 0.92 (0.87-0.97) | - | 0.92 (0.87-0.97) | 0.91 (0.86-0.97) | 0.91 (0.86-0.97) |
| Population Density (z-standardised) | - | - | 1.04 (0.98-1.10) | - | 1.06 (0.99-1.12) | 1.06 (0.99-1.13) |
| Cannabis prevalence (per 100% population increase) | - | - | - | 1.41 (1.16-1.72) | 1.41 (1.16-1.72) | - |

RR: relative risk; SFI: social fragmentation index.

^a^ Models 2-6 also included a random walk prior for the index of multiple deprivation (IMD).

Supplemental Table 7. Summary of predicted and observed counts of new FEP cases in 2017 for England, by age group, sex, and ethnicity (Models 1-6)^a^.

|  | **Observed** | **Model 1** | **Model 2** | **Model 3** | **Model 4** | **Model 5** | **Model 6** |
| --- | --- | --- | --- | --- | --- | --- | --- |
|  | **Cases** | **Cases (95% interval)** | **Cases (95% interval)** | **Cases (95% interval)** | **Cases (95% interval)** | **Cases (95% interval)** | **Cases (95% interval)** |
| **Total (age-matched)** | 8038 | 8984 (8497-9460) | 8137 (7642-8637) | 8362 (7907-8851) | 8112 (7623-8597) | 8187 (7722-8707) | 8205 (7716-8763) |
| **Age** |  |  |  |  |  |  |  |
| 16-35 | 5919 | 5832 (5535-6153) | 5398 (5086-5719) | 5579 (5265-5921) | 5378 (5078-5695) | 5446 (5120-5786) | 5452 (5141-5814) |
| 36-64 | 2119 | 3151 (2828-3518) | 2740 (2447-3068) | 2783 (2486-3108) | 2734 (2424-3053) | 2741 (2440-3049) | 2753 (2443-3105) |
| **Sex** |  |  |  |  |  |  |  |
| Female | 3240 | 3538 (3274-3847) | 3181 (2894-3466) | 3259 (2977-3535) | 3178 (2899-3467) | 3200 (2924-3490) | 3200 (2909-3490) |
| Male | 4790 | 5445 (5105-5835) | 4957 (4582-5317) | 5103 (4755-5467) | 4934 (4597-5284) | 4987 (4633-5357) | 5005 (4641-5413) |
| Unknown | 10 |  |  |  |  |  |  |
| **Ethnicity** |  |  |  |  |  |  |  |
| White British, Irish & Traveller | 4684 | 4937 (4579-5299) | 4582 (4236-4939) | 4667 (4330-5006) | 4570 (4235-4907) | 4605 (4284-4966) | 4616 (4282-5005) |
| White Other | 531 | 873 (747-1021) | 769 (652-907) | 821 (693-977) | 767 (652-897) | 785 (666-923) | 788 (665-932) |
| Black Caribbean | 370 | 645 (563-734) | 541 (467-623) | 561 (483-649) | 538 (471-619) | 543 (471-633) | 545 (473-629) |
| Black African | 323 | 710 (611-830) | 597 (501-704) | 620 (530-722) | 593 (496-688) | 593 (499-690) | 593 (504-690) |
| Indian | 144 | 322 (231-431) | 284 (203-380) | 287 (211-385) | 281 (202-386) | 283 (198-390) | 282 (199-387) |
| Pakistani | 237 | 379 (277-498) | 371 (267-494) | 362 (266-485) | 370 (272-505) | 369 (263-489) | 368 (266-495) |
| Bangladeshi | 109 | 159 (125-199) | 125 (98-154) | 130 (102-164) | 125 (96-156) | 123 (96-156) | 124 (99-157) |
| Mixed | 249 | 534 (411-687) | 489 (381-619) | 515 (399-666) | 489 (368-615) | 500 (378-640) | 501 (384-626) |
| Other | 570 | 425 (335-542) | 380 (296-475) | 399 (305-510) | 380 (295-476) | 385 (298-488) | 388 (297-495) |
| Unknown | 820 |  |  |  |  |  |  |

^a^ Predicted data were age-matched to observed MHSDS data, according to the corresponding age range served by each CCG as reported in the EIPN national audit for the same period (see Supplemental Materials, section 7 for further details). Not all CCGs served the entire population aged 16-64 years old, so the figures in this table should not be interpreted as total predicted cases in England for a given stratum. For total predicted caseload sizes in England, for 2019-2025, see Supplemental Table 10.

Supplemental Table 8. Summary of differences between predicted and observed total counts of new FEP cases in England, by age, sex, and ethnicity (Models 1-6).

|  | **Difference, n (%)** | | | | | |
| --- | --- | --- | --- | --- | --- | --- |
|  | **Model 1** | **Model 2** | **Model 3** | **Model 4** | **Model 5** | **Model 6** |
| **Total** | 946 (11.8) | 99 (1.2) | 324 (4.0) | 74 (0.9) | 149 (1.9) | 167 (2.1) |
| **Age** |  |  |  |  |  |  |
| 16-35 | -87 (-1.5) | -521 (-8.8) | -340 (-5.7) | -541 (-9.2) | -473 (-8.0) | -467 (-7.9) |
| 36-64 | 1032 (48.8) | 621 (29.3) | 664 (31.4) | 615 (29.0) | 622 (29.4) | 634 (29.90) |
| **Sex** |  |  |  |  |  |  |
| Female | 298 (9.2) | -59 (-1.8) | 19 (0.6) | -62 (-1.9) | -40 (-1.2) | -40 (-1.2) |
| Male | 655 (13.7) | 167 (3.5) | 313 (6.5) | 144 (3.0) | 197 (4.1) | 215 (4.5) |
| **Ethnicity^a^** |  |  |  |  |  |  |
| White British, Irish & Traveller | 253 (5.4) | -102 (-2.2) | -17 (-0.4) | -114 (-2.4) | -79 (-1.7) | -68 (-1.5) |
| White Other | 342 (64.4) | 238 (44.8) | 290 (54.6) | 236 (44.4) | 254 (48.0) | 257 (48.4) |
| Black Caribbean | 275 (74.3) | 171 (46.1) | 191 (51.7) | 168 (45.4) | 173 (46.8) | 175 (47.3) |
| Black African | 387 (119.9) | 274 (84.7) | 297 (92.0) | 270 (83.5) | 270 (83.5) | 270 (83.6) |
| Indian | 178 (123.3) | 140 (96.9) | 143 (99.2) | 137 (95.4) | 139 (96.5) | 138 (95.7) |
| Pakistani | 142 (60.1) | 134 (56.6) | 125 (52.9) | 133 (56.0) | 132 (55.8) | 131 (55.1) |
| Bangladeshi | 50 (45.6) | 16 (14.9) | 21 (19.1) | 16 (14.6) | 14 (13.3) | 15 (14.1) |
| Mixed | 285 (114.5) | 240 (96.4) | 266 (107.0) | 240 (96.4) | 251 (100.7) | 252 (101.0) |
| Other | -145 (-25.4) | -190 (-33.3) | -171 (-30.1) | -190 (-33.4) | -185 (-32.5) | -182 (-31.9) |

^a^ Error differences partially driven by 820 observed *probable FEP* cases missing ethnicity in the MHSDS; see Supplemental Table 9 for sensitivity analyses on possible imputation scenarios for this missing data.

Supplemental Table 9. Sensitivity analysis to evaluate predictive accuracy of Model 4 under different imputation scenarios to account for missing observed FEP data by ethnicity.

|  | **Model 4** | | **Observed** | | | **Scenario #1^a^** | | | **Scenario #2^b^** | | | **Scenario #3^c^** | | |
| --- | --- | --- | --- | --- | --- | --- | --- | --- | --- | --- | --- | --- | --- | --- |
|  | **Predicted cases** | **%** | **Actual**  **cases** | **%** | **Difference^d^**  **n (%)** | **Actual**  **cases** | **%** | **Difference^d^**  **n (%)** | **Actual**  **cases** | **%** | **Difference^d^**  **n (%)** | **Actual**  **cases** | **%** | **Difference^d^**  **n (%)** |
| White British, Irish & Traveller | 4570 | 50.9 | 4684 | 58.3 | -114 (-2.4) | 5504 | 68.5 | -934 (-17.0) | 4684 | 58.3 | -114 (-2.4) | 5146 | 64.0 | -576 (-11.2) |
| White Other | 767 | 8.5 | 531 | 6.6 | 236 (44.4) | 531 | 6.8 | 236 (44.4) | 708 | 8.8 | 58 (8.3) | 609 | 7.6 | 158 (26.0) |
| Black Caribbean | 538 | 6.0 | 370 | 4.6 | 168 (45.4) | 370 | 4.9 | 168 (45.4) | 495 | 6.2 | 43 (8.8) | 424 | 5.3 | 114 (26.9) |
| Black African | 593 | 6.6 | 323 | 4.0 | 270 (83.5) | 323 | 4.2 | 270 (83.5) | 460 | 5.7 | 133 (28.8) | 383 | 4.8 | 210 (54.8) |
| Indian | 281 | 3.1 | 144 | 1.8 | 137 (95.4) | 144 | 1.9 | 137 (95.4) | 209 | 2.6 | 72 (34.4) | 172 | 2.1 | 109 (63.2) |
| Pakistani | 370 | 4.1 | 237 | 3.0 | 133 (56.0) | 237 | 3.3 | 133 (56.0) | 323 | 4.0 | 47 (14.7) | 274 | 3.4 | 96 (35.0) |
| Bangladeshi | 125 | 1.4 | 109 | 1.4 | 16 (14.6) | 109 | 1.4 | 16 (14.6) | 138 | 1.7 | -13 (-9.4) | 122 | 1.5 | 3 (2.5) |
| Mixed | 489 | 5.4 | 249 | 3.1 | 240 (96.4) | 249 | 3.2 | 240 (96.4) | 362 | 4.5 | 127 (35.0) | 298 | 3.7 | 191 (64.0) |
| Other | 380 | 4.2 | 570 | 7.1 | -190 (-33.4) | 570 | 7.4 | -190 (-33.4) | 658 | 8.2 | -278 (-42.2) | 608 | 7.6 | -228 (-37.5) |
| Missing |  |  | 820 | 10.2 | 896 (11.1) | 0 | 0.0 |  | 0 | 0.0 |  | 0 | 0.0 |  |

^a^ Missing observed data assigned to “white British, Irish & Traveller” category.

^b^ Missing observed data assigned across all ethnic minority categories (i.e. except “white British, Irish & Traveller”), proportional to the distribution of the predicted FEP caseload in these categories. Scenario #2 led to the lowest error between observed and predicted *probable FEP* caseloads.

^c^ Missing observed data assigned across all ethnic groups, proportional to the predicted FEP caseload in these categories.

^d^ Difference between predicted minus observed *probable FEP* caseload. Percentage is expressed as a proportion of the observed caseload.

Supplemental Table 10. Predicted referred, assessed treated and probable FEP caseloads in England, by age group between 2019-2025

| **Stratum^a^** | **2019** | **2020** | **2021** | **2022** | **2023** | **2024** | **2025** | **% Change^b^** |
| --- | --- | --- | --- | --- | --- | --- | --- | --- |
|  | **N (95% interval)** | **N (95% interval)** | **N (95% interval)** | **N (95% interval)** | **N (95% interval)** | **N (95% interval)** | **N (95% interval)** | **% (95% interval)** |
| **16-64** |  |  |  |  |  |  |  |  |
| Referred | 24,286  (22,752 to 25,766) | 24,499  (22930 to 25991) | 24,715  (23,156 to 26,220) | 24,967  (23,417 to 26487) | 25,225  (23,658 to 26,761) | 25,508  (23,930 to 27,069) | 25,782  (24,189 to 27,349) | 6.5  (-2.8 to 16.8) |
| Accepted | 21,841  (20462 to 23172) | 22,033  (20,622 to 23,375) | 22,227  (20,825 to 23,581) | 22,454  (21,059 to 23,821) | 22,685  (21,277 to 24,067) | 22,940  (21,521 to 24,344) | 23,187  (21,754 to 24,596) | 6.3  (-2.8 to 15.7) |
| Treated | 10,425  (9,766 to 11,060) | 10,516  (9,843 to 11,157) | 10,609  (9,940 to 11,255) | 10,717  (10,052 to 11,370) | 10,828  (10,155 to 11,487) | 10,950  (10,272 to 11,620) | 11,067  (10,383 to 11,740) | 6.2  (-3.2 to 15.6) |
| Probable | 8,987  (8,419 to 9,535) | 9,066  (8,485 to 9,618) | 9,146  (8,569 to 9,703) | 9,239  (8,665 to 9,801) | 9,334  (8,755 to 9,903) | 9,439  (8,855 to 10,017) | 9,541  (8,951 to 10,120) | 6.4  (-3.0 to 15.4) |
| Population at-risk | 35,693,921 | 35,913,843 | 36,123,306 | 36,338,961 | 36,544,701 | 36,758,382 | 36,949,205 | 3.5 (-) |
| **16-35** |  |  |  |  |  |  |  |  |
| Referred | 15,569  (14,699 to 16,486) | 15,666  (14,791 to 16,593) | 15,772  (14,888 to 16,697) | 15,919  (15,023 to 16,857) | 16,067  (15,160 to 17,006) | 16,242  (15,323 to 17,192) | 16,403  (15,471 to 17,363) | 5.2  (-3.6 to 14.1) |
| Accepted | 14,002  (13,219 to 14,827) | 14,089  (13,302 to 14,922) | 14,184  (13,390 to 15,016) | 14,317  (13,511 to 15,160) | 14,449  (13,634 to 15,294) | 14,607  (13,781 to 15,462) | 14,752  (13,914 to 15,615) | 5.5  (-3.1 to 14.5) |
| Treated | 6,683  (6,309 to 7,077) | 6,725  (6,349 to 7,123) | 6,770  (6,391 to 7,167) | 6,833  (6,449 to 7,236) | 6,897  (6,508 to 7,300) | 6,972  (6,578 to 7,380) | 7,041  (6,641 to 7,453) | 5.6  (-2.8 to 14.6) |
| Probable | 5,761  (5,439 to 6,101) | 5,797  (5,473 to 6,140) | 5,836  (5,509 to 6,179) | 5,891  (5,559 to 6,238) | 5,945  (5,610 to 6,293) | 6,010  (5,670 to 6,362) | 6,070  (5,725 to 6,425) | 5.5  (-2.7 to 13.9) |
| Population at-risk | 14,961,038 | 15,010,547 | 15,061,784 | 15,135,691 | 15,193,574 | 15,265,728 | 15,307,915 | 2.3 (-) |
| **36-64** |  |  |  |  |  |  |  |  |
| Referred | 8,717  (7,750 to 9723) | 8,833  (7,849 to 9,863) | 8,944  (7,946 to 9,983) | 9,048  (8,033 to 10,103) | 9,158  (8,122 to 10,231) | 9,266  (8,213 to 10,356) | 9,379  (8,309 to 10,474) | 7.9  (-7.9 to 27.8) |
| Accepted | 7,840  (6,970 to 8,744) | 7,944  (7,059 to 8,870) | 8,043  (7,146 to 8,978) | 8,137  (7,225 to 9,086) | 8,236  (7,304 to 9,201) | 8,333  (7,386 to 9,313) | 8,435  (7,473 to 9,420) | 8.1  (-9.4 to 27.6) |
| Treated | 3,742  (3,327 to 4,174) | 3,792  (3,369 to 4,234) | 3,839  (3,411 to 4,285) | 3,884  (3,448 to 4,337) | 3,931  (3,486 to 4,392) | 3,978  (3,525 to 4,445) | 4,026  (3,567 to 4,496) | 7.8  (-9.8 to 24.7) |
| Probable | 3,226  (2,868 to 3,598) | 3,269  (2,905 to 3,650) | 3,310  (2,940 to 3,694) | 3,348  (2,973 to 3,739) | 3,389  (3,005 to 3,786) | 3,429  (3,039 to 3,832) | 3,471  (3,075 to 3,876) | 8.4  (-7.4 to 26.8) |
| Population at-risk | 20,732,883 | 20,903,296 | 21,061,522 | 21,203,270 | 21,351,127 | 21,492,654 | 21,641,291 | 4.4 (-) |

^a^ Referred: Referred to EIP services; Accepted: Accepted for EIP assessment; Treated: Treated by EIP services; Probable: Probable FEP. Based on PsyMaptic-A model 4.

^b^ Percentage change from 2019 to 2025; 95% intervals were not produced for the population at-risk

Supplemental Table 11. Twenty highest and lowest incidence rates of predicted FEP and treated caseloads in EIP services in 2020 at CCG level.

|  | **Highest^a^** | | | | | | **Lowest^a^** | | | | |
| --- | --- | --- | --- | --- | --- | --- | --- | --- | --- | --- | --- |
|  |  | **New treated caseload per annum** | | **New FEP caseload per annum** | |  | | **New treated caseload per annum** | | **New FEP caseload per annum** | |
|  | **CCG** | **Rate^b^** | **Count^c^** | **Rate^b^** | **Count^c^** | **CCG** | | **Rate^b^** | **Count^c^** | **Rate^b^** | **Count^c^** |
| 1 | Bradford City | 73.7 | 53 | 63.6 | 46 | Horsham & Mid Sussex | | 18.5 | 26 | 16.0 | 23 |
| 2 | Sandwell & West Birmingham | 55.0 | 190 | 47.5 | 164 | Eastern Cheshire | | 18.8 | 22 | 16.0 | 19 |
| 3 | Newham | 54.1 | 145 | 46.6 | 125 | Rushcliffe | | 18.7 | 13 | 16.1 | 11 |
| 4 | Barking & Dagenham | 53.9 | 76 | 46.5 | 65 | Harrogate & Rural District | | 19.0 | 19 | 16.4 | 16 |
| 5 | Manchester | 50.4 | 210 | 43.5 | 181 | High Weald Lewes Havens | | 19.1 | 19 | 16.5 | 17 |
| 6 | City & Hackney | 48.9 | 99 | 42.1 | 86 | West Hampshire | | 19.1 | 64 | 17.1 | 55 |
| 7 | Lewisham | 48.5 | 108 | 41.8 | 93 | South Warwickshire | | 19.8 | 33 | 17.2 | 28 |
| 8 | Waltham Forest | 47.6 | 98 | 41.1 | 85 | Surrey Downs | | 19.9 | 36 | 17.3 | 31 |
| 9 | Southwark | 47.4 | 114 | 40.9 | 98 | Wiltshire | | 20.1 | 60 | 17.3 | 52 |
| 10 | Haringey | 47.1 | 97 | 40.6 | 84 | North Norfolk | | 20.1 | 20 | 17.3 | 17 |
| 11 | Enfield | 46.7 | 111 | 40.3 | 96 | Shropshire | | 20.1 | 38 | 17.4 | 33 |
| 12 | Luton | 46.7 | 75 | 40.2 | 64 | North Hampshire | | 20.1 | 28 | 17.4 | 25 |
| 13 | Tower Hamlets | 46.2 | 104 | 39.9 | 90 | South West Lincolnshire | | 20.2 | 15 | 17.4 | 13 |
| 14 | Birmingham & Solihull | 45.8 | 337 | 39.4 | 291 | Vale of York | | 20.2 | 46 | 17.4 | 40 |
| 15 | Brent | 45.4 | 113 | 39.1 | 97 | West Kent | | 20.2 | 59 | 17.4 | 51 |
| 16 | Croydon | 44.9 | 123 | 38.7 | 106 | Richmond | | 20.2 | 28 | 17.4 | 24 |
| 17 | Nottingham City | 44.9 | 103 | 38.7 | 89 | Fylde & Wyre | | 20.2 | 22 | 17.4 | 19 |
| 18 | Lambeth | 44.6 | 114 | 38.4 | 99 | Coastal West Sussex | | 20.3 | 59 | 17.5 | 51 |
| 19 | Bradford Districts | 43.4 | 98 | 37.4 | 84 | East Riding of Yorkshire | | 20.4 | 38 | 17.6 | 33 |
| 20 | Leicester City | 42.3 | 108 | 36.4 | 93 | Southport & Formby | | 20.4 | 13 | 17.6 | 11 |
| - | Wolverhampton | 42.2 | 70 | 36.4 | 60 | Surrey Health | | 20.4 | 12 | 17.6 | 11 |
| - | - | - | - | - | - | East Surrey | | 20.4 | 23 | 17.6 | 20 |

CCG: Clinical Commissioning Group; FEP: First Episode Psychosis.

^a^ Ranked by predicted rate of FEP caseloads.

^b^ Per 100,000 person-years; 95% interval not shown, but available from [www.psymaptic.org/](http://www.psymaptic.org/)

^c^ Rounded to nearest whole case; 95% interval not shown, but available from [www.psymaptic.org/](http://www.psymaptic.org/)

Supplemental Table 12. Twenty highest and lowest counts of predicted FEP and treated caseloads in EIP services in 2020 at CCG level.

|  | **Highest^a^** | | | | | **Lowest^a^** | | | | |
| --- | --- | --- | --- | --- | --- | --- | --- | --- | --- | --- |
|  |  | **New treated caseload per annum** | | **New FEP caseload per annum** | |  | **New treated caseload per annum** | | **New FEP caseload per annum** | |
|  | **CCG** | **Count^b^** | **Rate^c^** | **Count^b^** | **Rate^c^** | **CCG** | **Count^b^** | **Rate^c^** | **Count^b^** | **Rate^c^** |
| 1 | Birmingham & Solihull | 337 | 45.8 | 291 | 39.4 | Corby | 12 | 27.1 | 10 | 23.4 |
| 2 | Manchester | 210 | 50.4 | 181 | 43.5 | Surrey Health | 12 | 20.4 | 11 | 17.6 |
| 3 | Sandwell & West Birmingham | 190 | 55.0 | 164 | 47.5 | Vale Royal | 13 | 20.2 | 11 | 17.4 |
| 4 | Bristol, N. Somerset & S. Gloucs | 168 | 27.7 | 144 | 23.4 | Rushcliffe | 13 | 18.7 | 11 | 16.1 |
| 5 | Leeds | 166 | 32.2 | 143 | 27.8 | Southport & Formby | 13 | 20.4 | 11 | 17.6 |
| 6 | Devon | 163 | 23.0 | 141 | 19.9 | Wyre Forest | 13 | 22.7 | 11 | 19.6 |
| 7 | Derby & Derbyshire | 158 | 25.1 | 137 | 21.7 | South West Lincolnshire | 15 | 20.2 | 13 | 17.4 |
| 8 | Cambridgeshire & Peterborough | 155 | 25.7 | 134 | 22.1 | Nottingham West | 15 | 21.8 | 13 | 18.8 |
| 9 | Newham | 145 | 54.1 | 125 | 46.6 | Scarborough & Ryedale | 16 | 23.7 | 13 | 20.5 |
| 10 | Sheffield | 138 | 35.7 | 119 | 30.8 | Newark & Sherwood | 16 | 21.7 | 14 | 18.7 |
| 11 | Croydon | 123 | 44.9 | 106 | 38.7 | West Lancashire | 16 | 24.2 | 14 | 20.8 |
| 12 | Liverpool | 122 | 36.6 | 105 | 31.6 | Darlington | 16 | 24.2 | 14 | 20.8 |
| 13 | Lambeth | 114 | 44.6 | 99 | 38.4 | Bassetlaw | 16 | 24.0 | 15 | 20.7 |
| 14 | Southwark | 114 | 47.4 | 98 | 40.9 | Ashford | 18 | 22.9 | 16 | 19.7 |
| 15 | Brent | 113 | 45.4 | 97 | 39.1 | South Lincolnshire | 18 | 20.5 | 16 | 17.6 |
| 16 | Enfield | 111 | 46.7 | 96 | 40.3 | Isle of Wight | 19 | 22.3 | 16 | 19.3 |
| 17 | Leicester City | 108 | 42.3 | 93 | 36.4 | Swale | 19 | 26.9 | 16 | 23.2 |
| 18 | Lewisham | 108 | 48.5 | 93 | 41.8 | East Staffordshire | 19 | 23.3 | 16 | 20.1 |
| 19 | Nene | 105 | 25.5 | 91 | 22.0 | Cannock Chase | 19 | 22.2 | 16 | 19.1 |
| 20 | Tower Hamlets | 104 | 46.2 | 90 | 39.9 | Harrogate & Rural District | 19 | 19.0 | 16 | 16.4 |

CCG: Clinical Commissioning Group; FEP: First Episode Psychosis.

^a^ Ranked by predicted count of FEP caseloads; 95% interval not shown, but available from [www.psymaptic.org/](http://www.psymaptic.org/)

^b^ Rounded to nearest whole case; 95% interval not shown, but available from [www.psymaptic.org/](http://www.psymaptic.org/)

^c^ Per 100,000 person-years.
